# Effects of brain-computer interface-based rehabilitation on upper limb function, activities of daily living, and adverse events in patients with early stroke: a systematic review and meta-analysis

**DOI:** 10.3389/fnagi.2026.1737740

**Published:** 2026-03-24

**Authors:** Jing-Xue Wei, Yue-Mi Zhang, Shan-Shan Liang, Qi-Fu Li, Lang Huang, Yuan-Hong Dai, Zi-Ting Bi, Jing-Hua Xiao, Jian-Wen Xu, Yun-Shan Zhang

**Affiliations:** 1 Department of Rehabilitation Medicine, The First Affiliated Hospital of Guangxi Medical University, Nanning, China; 2 Department of Rehabilitation Medicine, The Guangxi Zhuang Autonomous Region Workers' Hospital, Nanning, China

**Keywords:** brain-computer interface, early stroke, upper extremity function, activities of daily living, adverse events, meta-analysis

## Abstract

**Background:**

Brain-computer interface-based rehabilitation represents an emerging neurorehabilitation approach for post-stroke motor recovery, yet its comprehensive effects on patients in the early phase after stroke, typically defined as within 3 months of onset, remain to be fully established. This systematic review and meta-analysis evaluated effects of this intervention on upper limb function, activities of daily living, and adverse events in individuals with early stroke.

**Methods:**

This study was conducted following PRISMA guidelines. Eligibility criteria were established for randomized controlled trials that encompassed: (1) participants were adults (≥18 years) within 3 months of stroke onset with upper limb motor impairment; (2) interventions included brain-computer interface-based rehabilitation, and (3) outcomes that measured upper limb function, activities of daily living, and adverse events. A systematic search was performed across PubMed, Embase, Cumulative Index to Nursing and Allied Health Literature, Cochrane Library, and China National Knowledge Infrastructure databases from their inception to August 23, 2025. Two independent reviewers assessed eligibility, compiled data, and appraised methodological rigor, potential bias, and reliability of the evidence. Meta-analysis was performed using RevMan 5.4 (Cochrane Collaboration, UK) and Stata 18 (StataCorp., USA), applying random-effects models to calculate mean differences (MD) or risk ratios (RR) with 95% confidence intervals (CI). Subgroup analyses, meta-regression, sensitivity analyses, and publication bias assessments were conducted where appropriate.

**Results:**

Nine studies involving 642 participants (212 females and 430 males) with a mean age of 59.77 years were included. For primary outcomes, brain-computer interface-based rehabilitation significantly improved upper limb function in patients with early stroke (MD = 5.02, 95% CI: 3.20, 6.84). Subgroup analyses revealed that no statistically significant differences were observed in the improvement of upper limb functionality among various patient demographics and intervention characteristics (all *p* > 0.05). For secondary outcomes, the pooled analysis suggested a potential improvement in activities of daily living with BCI-based rehabilitation (MD = 7.68, 95% CI: 0.32, 15.03), although this finding was accompanied by very high heterogeneity (*I*^2^ = 88%) and was not robust in sensitivity analyses, indicating low certainty of evidence. Subgroup analyses indicated that greater benefits might be observed in patients within 30 days after stroke onset and with intervention durations not exceeding 3 weeks. Regarding safety, preliminary data from a single study suggested no significant difference in adverse events between groups (*p* = 0.87), but the evidence base is currently insufficient to draw firm conclusions.

**Conclusions:**

Brain-computer interface-based rehabilitation is effective in improving upper limb motor function in patients with early stroke. Current evidence suggests a potential benefit for activities of daily living, but the evidence is of low certainty due to substantial heterogeneity and limited robustness. Subgroup analyses identified time from onset and intervention duration as potential effect modifiers for activities of daily living. Preliminary safety data from a single study are encouraging but insufficient to establish a safety profile. Further well-designed randomized controlled trials are needed to establish optimal brain-computer interface-based rehabilitation protocols, to confirm the potential benefit on activities of daily living with more robust evidence, and to evaluate long-term efficacy and safety.

**Systematic Review Registration:**

PROSPERO [Register number: CRD420251144151].

## 1 Introduction

Stroke poses a major challenge to global health due to its high incidence, significant disability rate, and substantial socioeconomic costs, collectively contributing to a significant disease burden (Katan and Luft, 2018; Phipps and Cronin, 2020). With the improvement of medical management of early stroke, defined as within 3 months of onset, the survival rate of early stroke patients has increased significantly, but most early stroke survivors still experience persistent functional impairments (Phipps and Cronin, 2020; Stinear et al., 2020; Hilkens et al., 2024). Upper limb dysfunction stands out as one of the most common and devastating sequelae of early stroke, with previous studies indicating that approximately 60–80% of patients experience this dysfunction during the early phase (Tang et al., 2024; Persson et al., 2012; Schwarz et al., 2019). Research suggests that the critical window for neurological functional recovery typically occurs within the first 3 months post-stroke, covering the acute phase (often ≤ 30 days) and the subacute phase (generally 1–3 months after onset), during which focused interventions can significantly enhance functional rehabilitation, while 15%-30% of individuals may still experience lasting permanent dysfunction beyond this critical timeframe (Zhang et al., 2024; Patel et al., 2019; Hebert et al., 2016). It is worth noting that the dynamic process of neural plasticity and functional recovery is not homogeneous within these 3 months (Chen et al., 2026). The acute phase is often characterized by more rapid spontaneous recovery and heightened cortical excitability, whereas the subsequent subacute phase involves more experience-dependent plastic remodeling (Chen et al., 2026; Qiao et al., 2023). This indicates that functional recovery of upper limb dysfunction is particularly critical within this early post-stroke period. Therefore, this study focuses on the effects of rehabilitation interventions in patients within 3 months of stroke onset, a timeframe that captures a period of heightened neuroplasticity and potential for meaningful recovery.

Early stroke patients primarily experience upper limb dysfunction due to disruption of neural pathways caused by brain injury (Dolganov and Karpova, 2019; Geng et al., 2022; Langhorne et al., 2011). Stroke lesions can trigger a series of pathophysiological reactions like neuroinflammation, oxidative stress, metabolic abnormalities, excitotoxicity and cell apoptosis, which not only directly damage key structures such as the motor cortex and corticospinal tract, but also interrupt the nerve conduction pathways related to upper limb movement, further affecting the integration and regulation of motor sensory input, and ultimately leading to decreased motor control ability and limited upper limb movement (Geng et al., 2022; Langhorne et al., 2011; Alsbrook et al., 2023; Iadecola and Anrather, 2011). Additionally, patients in the early stroke phase often develop secondary issues such as muscle atrophy, joint contractures, and reduced activity, which can lead to abnormal movement patterns, altered muscle tone, and diminished ability to execute functional tasks, thereby exacerbating upper limb dysfunction (Langhorne et al., 2011; Pollock et al., 2014). This dysfunction presents unique challenges to both patients and clinicians. For patients, it directly impairs voluntary movement, fine motor skills, and bimanual coordination, which severely restricts independence in daily activities, limits social participation, and reduces overall quality of life, leading to increased reliance on care (Patel et al., 2019; Geng et al., 2022; Langhorne et al., 2011). For healthcare professionals, managing this condition demands close functional monitoring and personalized interventions, adding to clinical workloads and requiring sustained resources to address sequelae like spasticity and pain while preventing functional decline (Patel et al., 2019; Langhorne et al., 2011; Pollock et al., 2014). Overall, upper limb dysfunction not only delays the overall recovery process but may also lead to long-term functional impairment, thereby significantly increasing the patient's risk of disability (Langhorne et al., 2011; Pollock et al., 2014). Therefore, seeking effective rehabilitation interventions to enhance upper limb function and promote overall recovery in early stroke patients is of considerable clinical importance.

Conventional rehabilitation therapies like physical and occupational therapy primarily rely on task-oriented training, muscle strengthening, and compensatory strategies to promote functional recovery, yet they exhibit significant limitations in patients with severe motor impairments, particularly during the early stages of stroke (Hebert et al., 2016; Langhorne et al., 2011; Roesner et al., 2024). Since these methods depend heavily on residual motor function, they often fail to deliver the necessary repetitive practice when voluntary movement is minimal, and patients frequently struggle to maintain adequate training intensity due to fatigue or diminished motivation (Ambrosini et al., 2021; Lo et al., 2024). More critically, traditional rehabilitation therapies are generally unable to precisely activate impaired motor neural circuits or effectively modulate cortical excitability, thereby hindering the induction of beneficial neuroplastic changes (Sebastián-Romagosa et al., 2020; Li et al., 2025). Moreover, such programs tend to adopt standardized protocols that lack individualization, making it difficult to adapt to dynamic neural states during recovery, limiting the potential for neural remodeling, and resulting in particularly poor outcomes for fine motor skills and coordination training (Sebastián-Romagosa et al., 2020; Xie et al., 2022; Ren et al., 2024). Therefore, there is an urgent need to explore innovative rehabilitation strategies that can make up for these shortcomings.

Brain-computer interface (BCI) is an emerging neuromodulation technology, which decodes the patient's motor imagery (MI) or other neural activity intentions in real time and drives external devices, such as functional electrical stimulation (FES), soft robotic gloves or virtual reality tasks to provide immediate, closed-loop sensorimotor feedback, thereby promoting motor function reconstruction (Lo et al., 2024; Li et al., 2025; Ren et al., 2024). The core physiological mechanism is based on the Hebbian plasticity principle, utilizing repetitive task-specific brain activation training to enhance functional reorganization between damaged brain regions and motor networks, promoting coordinated activation across bilateral hemispheres, thereby inducing neuroplastic changes conducive to functional recovery (Lo et al., 2024; Xie et al., 2022; Ren et al., 2024). Building upon this technological and physiological foundation, BCI-based rehabilitation is conceptualized as a standardized neurorehabilitation treatment program that integrates an active, closed-loop BCI system (Ma et al., 2025). Its core feature is the establishment of an intention–decoding–feedback reinforcement learning loop via the BCI system, which translates the patient's actively generated motor intention (e.g., MI) in real time into multimodal sensorimotor feedback, such as completing movements via FES or robotic assistance (Ma et al., 2025; Wang et al., 2025). This approach emphasizes that the patient's active participation and effort are pivotal for driving therapeutic neuroplasticity, with the fundamental aim of strengthening or reconstructing impaired motor control pathways through high-intensity, task-specific, and intention-synchronized repetitive training (Wang et al., 2025). It thus differs from conventional rehabilitation techniques that provide primarily passive assistance or open-loop stimulation, representing a more interactive and potentially individualized strategy. Current clinical studies indicate that BCI-based rehabilitation demonstrates a positive effect on improving upper limb function in stroke patients (Brunner et al., 2024; Zanona et al., 2023; Wu et al., 2020), yet evidence regarding its impact on early stroke patients remains unclear. Therefore, it is necessary to conduct a systematic review of existing clinical studies to evaluate the efficacy and safety of BCI-based rehabilitation in these specific patients.

In recent years, the number of systematic reviews and meta-analyses on the efficacy of BCI in stroke rehabilitation has increased. Existing evidence shows that BCI-based rehabilitation can effectively improve upper limb function (Lo et al., 2024; Li et al., 2025; Xie et al., 2022; Ren et al., 2024; Baniqued et al., 2021; Carvalho et al., 2019; Peng et al., 2022; Yang et al., 2021; Shou et al., 2023; Nojima et al., 2022) and daily living activities (Li et al., 2025; Xie et al., 2022; Peng et al., 2022; Shou et al., 2023) in stroke patients. Notably, only one meta-analysis has specifically assessed the safety of BCI-based rehabilitation in stroke, reporting no significant adverse effects (Xie et al., 2022). However, these systematic reviews did not separately examine the effects of BCI-based rehabilitation on early stroke patients, instead including studies with mixed cohorts of both early and chronic stroke participants. This approach may obscure phase-specific treatment effects, as the recovery trajectory and responsiveness to intervention can differ substantially between early and chronic phases. Combining these populations may lead to heterogeneity in results and unclear conclusions, making it difficult to reveal the potential therapeutic advantages of BCI-based rehabilitation during the critical early post-stroke period. Therefore, conducting a systematic review and meta-analysis to comprehensively evaluate the effects of BCI-based rehabilitation in early-stage stroke holds significant clinical importance and research value. Such a review would focus specifically on this phase and explore potential effect modifiers, such as time since stroke onset.

Thus, the objectives of this systematic review and meta-analysis were to evaluate the effects of BCI-based rehabilitation on upper limb function, activities of daily living, and adverse events in patients with early stroke.

## 2 Methods

This meta-analysis was performed in accordance with the guidelines established by the Preferred Reporting Items for Systematic Reviews and Meta-Analyses (PRISMA) (Page et al., 2021).

### 2.1 Eligibility criteria

The criteria for inclusion were established in accordance with the Population-Interventions-Comparison-Outcomes of interest-Study design (PICOS) framework. Specifically, it consisted of the following components: (1) participants were adults (≥18 years) within 3 months of stroke onset with upper limb motor impairment; (2) the intervention group (IG) must receive BCI-based rehabilitation, which includes specific protocols for training that utilize BCI technology to facilitate upper limb movement; (3) the control group (CG) receive either conventional rehabilitation programs without BCI-based upper limb functional rehabilitation or a placebo intervention that mimics BCI without providing actual therapeutic benefits; (4) the primary outcome should be alterations in upper limb function evaluated by the Fugl-Meyer Assessment (FMA); secondary outcomes may include activities of daily living evaluated through the Modified Barthel Index (MBI) or the Functional Independence Measure (FIM), alongside the occurrence of adverse events associated with the intervention, including but not limited to discomfort, injury, or any serious adverse events; (5) randomized controlled trials (RCTs). The exclusion criteria were delineated as follows: (1) abstracts, observational studies, qualitative studies, reviews, meta-analyses, case reports, letters, protocols, or studies not published in peer-reviewed journals; (2) duplicate or inaccessible full-text studies; (3) studies involving participants with severe cognitive impairments or other neurological conditions that may confound the results related to upper limb motor impairment; (4) studies lacking adequate specifics regarding the intervention, such as duration, frequency, and intensity; (5) studies failing to report the primary outcome; (6) studies with insufficient data for effect size (ES) and 95% confidence interval (CI); (7) studies exhibiting inadequate methodological rigor, characterized by a Physiotherapy Evidence Database (PEDro) score falling below 6 (Zhang et al., 2024; Fabero-Garrido et al., 2022; Wood et al., 2008).

### 2.2 Information sources

A thorough literature search was performed across five electronic databases, namely PubMed, Embase, Cumulative Index to Nursing and Allied Health Literature (CINAHL), Cochrane Library, and China National Knowledge Infrastructure (CNKI) databases, spanning from their inception until August 23, 2025. To guarantee exhaustive coverage, supplementary resources were also investigated, including clinical trial registries (ClinicalTrials.gov), websites (Google), and reference lists from all retained articles.

### 2.3 Search strategy

The search strategy was developed based on the predefined PICOS framework. To effectively capture the core concepts, keywords and associated terms were identified for each key component: (1) “acute stroke,” “sub-acute stroke,” “early stroke,” “cerebrovascular accident,” “stroke,” “brain vascular accident,” “brain attack,” “cerebral stroke,” “apoplexy,” “hemiplegia,” “CVA”; (2) “brain-computer interface,” “BCI,” “brain-machine interface,” “BMI,” “neural interface,” “neurotechnology”; (3) “upper limb function,” “arm function,” “upper extremity function,” “upper limb mobility,” “Fugl-Meyer Assessment,” “FMA,” “activities of daily living,” “ADL,” “Modified Barthel Index,” “MBI,” “Functional Independence Measure,” “FIM,” “adverse events,” “adverse effects,” “side effects,” “untoward effects,” “harmful effects,” “complications”; (4) “random^*^ control^*^ trials.” Boolean operators were systematically utilized to integrate these concept blocks, employing the OR operator to connect related concepts and the AND operator to link different concepts together. No language restrictions applied during the search process. After the initial search of the primary database, a supplementary manual search was performed across the additional resources mentioned above to guarantee the retrieval of comprehensive literature. As an example, the complete and reproducible search strategy for PubMed is presented below. Strategies for the other databases are available in Supplementary material.

(stroke [MeSH Terms] OR stroke [Text Word] OR acute stroke [Text Word] OR sub-acute stroke [Text Word] OR early stroke [Text Word] OR cerebrovascular accident [Text Word] OR brain vascular accident [Text Word] OR brain attack [Text Word] OR cerebral stroke [Text Word] OR apoplexy [Text Word] OR hemiplegia [Text Word] OR CVA [Text Word]) AND (brain-computer interface [MeSH Terms] OR brain-computer interface [Text Word] OR BCI [Text Word] OR brain-machine interface [Text Word] OR BMI [Text Word] OR neural interface [Text Word] OR neurotechnology [Text Word]) AND (upper limb function [Text Word] OR arm function [Text Word] OR upper extremity function [Text Word] OR upper limb mobility [Text Word] OR Fugl-Meyer Assessment [Text Word] OR FMA [Text Word] OR activities of daily living [Text Word] OR ADL [Text Word] OR Modified Barthel Index [Text Word] OR MBI [Text Word] OR Functional Independence Measure [Text Word] OR FIM [Text Word] OR adverse events [Text Word] OR adverse effects [Text Word] OR side effects [Text Word] OR untoward effects [Text Word] OR harmful effects [Text Word] OR complications [Text Word]) AND (randomized controlled trial [Publication Type]).

### 2.4 Selection process

All identified records from databases and supplementary sources were imported into EndNote 20 (Clarivate Analytics, UK) for duplicate removal. To enhance the efficiency of initial screening, a semi-automated step was implemented prior to manual review. After duplicate removal, all bibliographic records were exported to a spreadsheet (Microsoft Excel). A standardized list of exclusion keywords (e.g., review, protocol, and terms clearly unrelated to stroke or BCI-based rehabilitation) was applied to titles and abstracts. This permitted the rapid exclusion of records that were manifestly irrelevant. Two independent reviewers (JX and YM) then conducted a formal, manual screening of titles and abstracts against the predefined eligibility criteria, excluding those that failed to qualify. Full texts of potentially relevant articles were retrieved and independently assessed for inclusion by the same reviewers. The reviewers ultimately engaged in direct dialogue and meticulous proofreading of the final included studies. Any discrepancies during the selection process were resolved through discussion or by consulting a third reviewer (SS) when consensus could not be reached. The entire selection process was documented using a PRISMA flow diagram, which visually represented the number of records identified, excluded, and included at each stage, along with specific reasons for exclusion. This approach ensured transparency and clarity in the selection methodology.

### 2.5 Data collection process

A standardized, pre-piloted data extraction form was developed in Microsoft Excel to ensure systematic and consistent data collection. Two reviewers (QF and LH) independently extracted data from each included study. The extracted data encompassed the following key domains: study characteristics (authors and year), participant demographics (sample size, age, sex, time since stroke onset, body mass index, and baseline function score), interventional specifics (type, intensity, frequency, duration, and supervision), control composition, outcome parameters, and other relevant data as recommended by the Cochrane Handbook for Systematic Reviews of Interventions (Cumpston et al., 2019). If necessary, the authors of the primary studies would be approached by the investigator (YH) via email to request missing or unclear data. Any inconsistencies in the gathered data were corrected through mutual agreement or by seeking the expertise of a third-party reviewer (ZT).

### 2.6 Quality assessment

The methodological evaluation integrated three complementary tools to comprehensively assess study validity. Methodological quality of included RCTs was assessed using the PEDro scale, which is a well-validated and widely adopted tool for assessing methodological quality in physiotherapy research and is frequently employed in systematic reviews and meta-analyses (Cashin and McAuley, 2020; Moseley et al., 2019). This 11-item instrument evaluates key methodological dimensions: one item addresses external validity (eligibility criteria), eight items assess risk of bias (including random allocation, concealed allocation, baseline comparability, blinding of participants/therapists/assessors, adequate follow-up, and intention-to-treat analysis), and two items evaluate statistical reporting completeness (between-group comparisons and measures of variability) (Moseley et al., 2019). Scored on a 0–10 scale (excluding the first eligibility item), higher scores indicate superior methodological rigor (Maher et al., 2003). Based on established thresholds, studies are quality-graded as excellent (9–10), good (6–8), fair (4–5), or poor (< 4) (Gonzalez et al., 2018). This research established a methodological quality criterion for inclusion by requiring a PEDro score exceeding 6. The pre-specified threshold was established to include studies with at least good methodological quality, thereby enhancing the internal validity and reliability of the pooled effect estimates and allowing for a more accurate assessment of the intervention effect. This approach aligns with widely adopted practices in high-quality systematic reviews within the field of neurorehabilitation, striking a reasonable balance between methodological rigor and evidence representativeness (Zhang et al., 2024; Dhariwal et al., 2025). Furthermore, given that BCI-based early stroke rehabilitation constitutes an emerging and rapidly evolving field, the methodological quality of initial studies may be heterogeneous. Studies with scores below this threshold typically have deficiencies in key areas of bias control, such as allocation concealment and assessor blinding, which may affect the authenticity of efficacy assessments. Therefore, from a methodological standpoint, prioritizing the synthesis of a more reliable evidence base is particularly crucial for interpreting therapeutic effects in this domain. Concurrently, the items of the Cochrane Risk of Bias tool were employed to evaluate the risk of bias in the included studies (Cumpston et al., 2019). All assessments were documented and visualized using Review Manager version 5.4 (Cochrane Collaboration, UK) (Cumpston et al., 2019). This tool assesses six key domains of bias: selection bias, performance bias, detection bias, attrition bias, reporting bias, and other biases. Each domain was judged as “low,” “high,” or “unclear” risk of bias based on specific criteria within the tool (Cumpston et al., 2019). Additionally, the certainty of evidence for all outcomes was graded as high, moderate, low, or very low using the Grading of Recommendations Assessment, Development and Evaluation (GRADE) framework through systematic evaluation of five key domains: serious risk of bias (based on PEDro scores < 6), inconsistency (*I*^2^ > 50%), indirectness of populations/interventions/outcomes, imprecision (95% CIs crossing clinical decision thresholds or optimal information size not met), and publication bias (Brignardello-Petersen and Guyatt, 2025; Gonzalez-Padilla and Dahm, 2021). Notably, each domain could be downgraded by up to two levels, and the baseline evidence quality was set as high for RCTs and as low for observational studies (Gonzalez-Padilla and Dahm, 2021; Zhang et al., 2019). Two independent reviewers (JH and JW) conducted all assessments using standardized protocols, with discrepancies resolved through consensus or third-reviewer arbitration (YS), and results were visualized through summary tables, graphs, and evidence profiles, ultimately forming a three-dimensional evaluation system of methodological quality, risk of bias, and strength of evidence.

### 2.7 Data synthesis and analysis

All data synthesis and analyses were performed using RevMan 5.4 (Cochrane Collaboration, UK) (Cumpston et al., 2019) and Stata 18 (StataCorp., USA) (StataCorp, 2023). Meta-analysis was only conducted when three or more RCTs reported data for a given outcome; otherwise, a descriptive synthesis was provided (Fabero-Garrido et al., 2022; Huang et al., 2025). A random-effects model was applied for all meta-analyses to account for potential clinical and methodological heterogeneity (Halme et al., 2023). Study heterogeneity was assessed using Cochran's Q statistic and the *I*^2^ index (Huedo-Medina et al., 2006). Interpretation of *I*^2^ followed Cochrane guidelines: 0%−40% (unimportant), 30%−60% (moderate), 50%-90% (substantial), 75%−100% (considerable) (Huedo-Medina et al., 2006). Statistically significant heterogeneity was defined as Q statistic *p* < 0.1 or *I*^2^ > 50% (Huedo-Medina et al., 2006). For continuous outcomes related to upper limb function (e.g., FMA scores), the mean difference (MD) from baseline to post-intervention and its standard deviation (SD) (i.e., change scores) were used for analysis. The pooled MD with 95% CI or standardized mean difference (SMD) with 95% CI was then calculated, depending on whether the same assessment scale was used across studies. For dichotomous outcomes (e.g., adverse events), risk ratios (RR) with 95% CI were computed. When studies reported median and interquartile range (IQR) without mean and SD, the CI calculation was derived by converting median to mean and IQR to SD using the formula SD=IQR/1.35, consistent with Cochrane methods for skewed data transformation (Cumpston et al., 2019). The specific studies and outcomes that required this data conversion were explicitly reported. Statistical significance was defined as *p* < 0.05 for all analyses.

To investigate possible sources of clinical heterogeneity, subgroup analyses and meta-regression assessments were conducted. Where sufficient data were available (Cumpston et al., 2019), subgroup analyses were conducted based on factors such as intervention duration, BCI type, and baseline severity. Meta-regression analyses exploring associations between the pooled ES and baseline covariates (sex distribution expressed as % female, mean patient age) were conducted when at least 10 studies reported primary outcomes, with subgroup analyses performed when study numbers were insufficient for meta-regression (Cumpston et al., 2019, 2022). Sensitivity analyses were performed to evaluate the robustness of the results through conducting leave-one-out analyses, which involve iteratively removing each study to assess its individual influence on the pooled ES. Publication bias was assessed visually using funnel plots and statistically using Egger's test if more than 10 studies were included in a meta-analysis (Cumpston et al., 2019).

## 3 Results

### 3.1 Study selection

**Figure 1:**
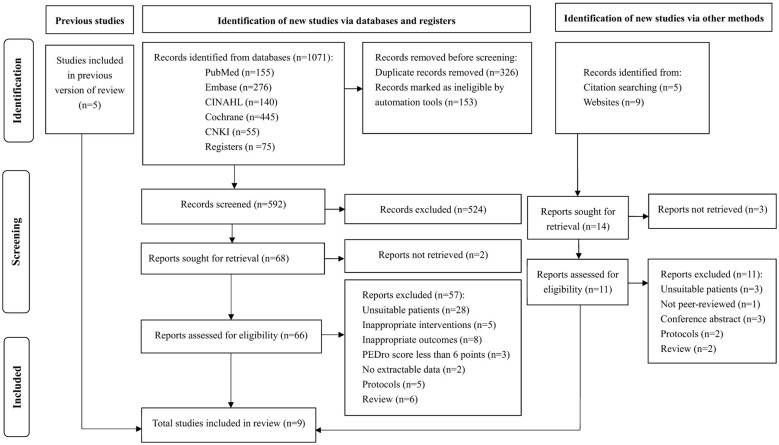
PRISMA search flow diagram.

The comprehensive search process identified a total of 1071 studies, including 155 studies from PubMed, 276 studies from Embase, 140 studies from CINAHL, 445 studies from Cochrane, and 55 studies from CNKI. Additionally, 75 studies were identified from clinical trial registers. After removing 326 duplicate records, a further 153 records were excluded by the automated keyword screening process, leaving 592 records for manual screening. Following a detailed evaluation of titles and abstracts, 524 records were excluded for failing to satisfy the inclusion criteria, resulting in 68 studies that were eligible for full-text retrieval. Unfortunately, the full texts of two reports could not be retrieved. In addition to database searches, 14 studies were identified through website and citation searches, though three reports were not retrievable. Thus, 77 studies were retrieved and assessed for eligibility. Ultimately, nine studies (He et al., 2025; Zhang et al., 2025; Ji et al., 2025; Hou et al., 2024; Liu et al., 2023; Liao et al., 2023; Dun et al., 2023; Wang et al., 2024, 2022) fulfilled the eligibility criteria and were incorporated into the systematic review and meta-analysis, while the remaining 68 studies were excluded due to factors such as unsuitable patient demographics, inappropriate interventions and outcomes, as well as a PEDro score of less than 6 points. Notably, five studies (Liu et al., 2023; Liao et al., 2023; Dun et al., 2023; Wang et al., 2024, 2022) that had been included in previous systematic reviews were also identified and retrieved through the independent search. These studies underwent the same rigorous screening and eligibility assessment as all newly identified studies. Figure 1 presents the PRISMA search flow diagram, which illustrates the rigorous selection process employed to ensure high-quality evidence for the analysis.

### 3.2 Study characteristics

The included nine studies (He et al., 2025; Zhang et al., 2025; Ji et al., 2025; Hou et al., 2024; Liu et al., 2023; Liao et al., 2023; Dun et al., 2023; Wang et al., 2024, 2022) were published between 2022 and 2025. Four additional studies (He et al., 2025; Zhang et al., 2025; Ji et al., 2025; Hou et al., 2024) published in 2024 and 2025 were identified, providing novel insights and emerging evidence in this field. To enable cross-study comparisons, Table 1 summarizes the demographic and baseline characteristics of participants from all included studies, whereas Table 2 provides details on intervention protocols, outcome measures, and key results.

**Table 1:** Participant characteristics across the included studies.

**Study**	**Sample size**	**Age (years)**	**Female (%)**	**Time from onset (days)**	**BMI (kg/m^2^)**	**Initial FMA-UL score**	**Sample size**	**Age (years)**	**Female (%)**	**Time from onset (days)**	**BMI (kg/m^2^)**	**Initial FMA-UL score**
	**IG**						**CG**					
(He et al. 2025)	25	58.60 ± 12.80	5 (20.0)	33.30 ± 18.50	NR	23.00 ± 25.93	23	58.30 ± 12.8	9 (39.1)	41.80 ± 19.10	NR	18.00 ± 13.33
(Zhang et al. 2025)	20	52.55 ± 11.08	7 (35.0)	27.45 ± 11.60	NR	NR	20	51.25 ± 9.79	10 (50.0)	22.70 ± 7.43	NR	NR
(Ji et al. 2025)	20	61.75 ± 10.35	8 (40.0)	49.50 ± 23.36	24.32 ± 2.66	22.00 ± 6.30	19	60.05 ± 14.35	4 (21.1)	42.42 ± 24.50	24.61 ± 2.51	21.00 ± 4.44
(Hou et al. 2024)	29	68.93 ± 5.20	16 (55.2)	42.30 ± 23.70	32.67 ± 2.50	NR	28	67.64 ± 5.20	13 (46.4)	46.80 ± 22.80	23.41 ± 2.10	NR
(Wang et al. 2024)	150	60.00 ± 11.11	40 (26.7)	15.00 ± 9.630	24.44 ± 3.39	28.00 ± 20.00	146	58.00 ± 10.37	31 (21.2)	13.00 ± 7.41	24.69 ± 3.06	31.00 ± 26.67
(Liu et al. 2023)	30	52.50 ± 10.59	8 (26.7)	18.50 ± 9.11	NR	NR	30	53.00 ± 15.56	11(36.7)	18.00 ± 9.78	NR	NR
(Liao et al. 2023)	20	61.50 ± 3.80	12 (60.0)	20.10 ± 2.20	NR	18.50 ± 6.46	20	61.00 ± 3.70	11 (55.0)	20.60 ± 2.40	NR	18.70 ± 6.23
(Dun et al. 2023)	12	53.47 ± 4.23	4 (33.3)	51.00 ± 12.00	NR	NR	10	61.00 ± 3.00	4 (36.4)	57.00 ± 15.00	NR	NR
(Wang et al. 2022)	20	69.05 ± 5.79	11 (55.0)	42.00 ± 25.80	23.84 ± 2.76	NR	20	67.25 ± 4.78	8 (40.0)	47.10 ± 23.10	23.61 ± 2.30	NR
Average	36	59.82 ± 8.33	12 (39.1)	33.24 ± 16.30	26.32 ± 2.83	22.88 ± 14.67	35	59.72 ± 8.84	11 (38.4)	34.38 ± 14.61	24.08 ± 2.49	22.18 ± 12.67

IG, Intervention group; CG, Control group; BMI, Body mass index; FMA, Fugl-Meyer Assessment; UL, Upper limb; NR, Not reported in the source trial. Data are mean ± standard deviation and percentages.

**Table 2:** Summary of intervention protocols and outcome results.

**Study**	**Intervention**	**Control**	**Outcome measures**	**Main results**
He et al. (2025)	Type: BCI-robot-MI-VF Intensity: fixed Frequency: 20 min/session, 1 session/day, 5 days/week Duration: 2 weeks Supervision: supervised by the therapist Conventional rehabilitation programs as CG	Conventional rehabilitation programs: acupuncture, physiotherapy therapy, occupational therapy and electronic biofeedback (30 min/day, 5 days/week, 2 weeks) A placebo intervention that simulates BCI without delivering genuine therapeutic advantages	Primary outcome: FMA Secondary outcomes: NR	The IG showed a significant improvement in the FMA-UL compared to the CG (*p* = 0.046)
(Zhang et al. 2025)	Type: BCI-FES-MI-VF Intensity: adjusted Frequency: 30 min/session, 1 session/day, 5 days/week Duration: 3 weeks Supervision: supervised by the therapist Conventional rehabilitation programs as CG	Conventional rehabilitation programs: conventional rehabilitation therapy, including exercise therapy and occupational therapy, combined with acupuncture (3 h/day, 5 days/week, 3 weeks), and conventional upper limb motor function training (30 min/session, 1 session/day, 5 days/week, 3 weeks)	Primary outcome: FMA Secondary outcomes: MBI	The IG demonstrated significantly higher scores on the FMA-UL and MBI assessments compared to the CG, with a statistically significant difference (*p* < 0.05)
Ji et al. (2025)	Type: BCI-robot-MI-VF Intensity: fixed Frequency: 20–25 min/day, 1 session/day, 5 days/week Duration: 4 weeks Supervision: supervised by the therapist Conventional rehabilitation programs as CG	Conventional rehabilitation programs: therapist guided active and passive limb exercises, task-oriented exercises, ADL training, and neuromuscular electrical stimulation (60 min/session, 5 days/week, 4 weeks), and upper limb motor function training with a soft robotic glove (20 min/session, 1 session/day, 5 days/week, 4 weeks)	Primary outcome: FMA Secondary outcomes: MBI	Compared with the CG, the FMA-UL of the IG was more significantly improved (*p* = 0.010), while there was no significant difference in the MBI (*p* = 0.065)
(Hou et al. 2024)	Type: BCI-FES-MI-VF Intensity: adjusted Frequency: 20 min/session, 1 session/day, 5 days/week Duration: 4 weeks Supervision: supervised by the therapist Conventional rehabilitation programs as CG	Conventional rehabilitation programs: motor therapy including rolling over, sitting up, gait training, and joint range of motion exercises (40 min/session, 1 session/day, 5 days/week, 4 weeks), and occupational therapy encompassing ADL training, hand function training, and cognitive training (30 min/session, 1 session/day, 5 days/week, 4 weeks) The CG also received neuromuscular electrical stimulation treatment targeting wrist dorsiflexor muscles separately (20 min/session, 1 session/day, 5 days/week, 4 weeks)	Primary outcome: FMA Secondary outcomes: NR	The IG showed a significant improvement in the FMA-UL compared to the CG (*p* < 0.05)
(Wang et al. 2024)	Type: BCI-FES-MI-VF Intensity: adjusted Frequency: 30 min/session, 1 session/day, 5 days/week Duration: 4 weeks Supervision: supervised by the therapist Conventional rehabilitation programs as CG	Conventional rehabilitation programs: motor therapy, occupational therapy, physical factor therapy, coordination, anti-spasticity training, acupuncture, and moxibustion therapy (30 min/session, 1 session/day, 5 days/week, 4 weeks)	Primary outcome: FMA Secondary outcomes: adverse events	The IG showed a significant improvement in the FMA-UL compared to the CG (*p* = 0.0045) The incidence of adverse events was similar between the IG (22.00%, 33/150) and the CG (21.23%, 31/146), with no significant difference (*p* = 0.87)
(Liu et al. 2023)	Type: BCI-FES-MI-VF Intensity: adjusted Frequency: 20 min/session, 1 session/day, 5 days/week Duration: 3 weeks Supervision: supervised by the therapist Conventional rehabilitation programs as CG	Conventional rehabilitation programs: therapist-guided motor therapy, occupational therapy, physical factor therapy, coordination/anti-spasticity training, acupuncture, and moxibustion (1 session/day, 5 days/week, 3 weeks) The CG also received FES (20 min/session, 1 session/day, 5 days/week, 3 weeks)	Primary outcome: FMA Secondary outcomes: MBI	The IG demonstrated significantly higher scores on the FMA-UL and MBI assessments compared to the CG, with a statistically significant difference (*p* < 0.001)
(Liao et al. 2023)	Type: BCI-FES-MI-VF Intensity: adjusted Frequency: 45–60 min/session, 1 session/day, 5 days/week Duration: 3 weeks Supervision: supervised by the therapist Conventional rehabilitation programs as CG	Conventional rehabilitation programs: balance training (standing/sitting), left of gravity transfer training, bed and chair transfer training, walking training, brace and walker-assisted training, and ADL training (grooming, mobility, dressing, and toilet) (45–60 min/session, 1 session/day, 5 days/week, 3 weeks)	Primary outcome: FMA Secondary outcomes: NR	The IG showed a significant improvement in the FMA-UL compared to the CG (*p* < 0.05)
(Dun et al. 2023)	Type: BCI-FES-MI-MF Intensity: adjusted Frequency: 20–30 min/session, 1 session/day, 5 days/week Duration: 8 weeks Supervision: supervised by the therapist Conventional rehabilitation programs as CG	Conventional rehabilitation programs: therapist-guided motor therapy, occupational therapy, physical factor therapy, and acupuncture (1 session/day, 5 days/week, 8 weeks)	Primary outcome: FMA Secondary outcomes: MBI	The IG demonstrated significantly higher scores on the FMA-UL and MBI assessments compared to the CG, with a statistically significant difference (*p* < 0.05)
(Wang et al. 2022)	Type: BCI-FES-MI-VF Intensity: adjusted Frequency: 20 min/session, 1 session/day, 5 days/week Duration: 4 weeks Supervision: supervised by the therapist Conventional rehabilitation programs as CG	Conventional rehabilitation programs: motor therapy, including turning-over/sitting-up training, walking function training, and joint function training (40 min/session, 1 session/day, 5 days/week, 4 weeks), occupational therapy, like hand function training, cognitive training, ADL training (30 min/session, 1 session/day, 5 days/week, 4 weeks)	Primary outcome: FMA Secondary outcomes: MBI	Compared with the CG, the FMA-UL of the IG was more significantly improved (*p* < 0.05), while there was no significant difference in the MBI (*p* > 0.05)

IG, Intervention group; CG, Control group; BCI, Brain-computer interface; MI, Motor imagery; FES, Functional electric stimulation; VF, Virtual feedback; MF, Mirror feedback; FMA, Fugl-Meyer Assessment; UL, Upper limb; ADL, Activities of daily living; MBI, Modified Barthel Index; NR, Not reported in the source trial.

#### 3.2.1 Participants

A total of 642 participants were included across the studies, with sample sizes ranging from 22 (Dun et al., 2023) to 296 (Wang et al., 2024). The overall mean age was 59.77 ± 8.59 years, and the sex distribution consisted of 212 females (33%) and 430 males (67%). All study participants were in the early stages of stroke, within 3 months of the onset of the stroke, but the specific time of stroke onset varied among those included in the study. The mean time from stroke onset was 33.81 ± 15.46 days. Regarding body mass index (BMI), most studies did not report this measure, while only four studies (Ji et al., 2025; Hou et al., 2024; Wang et al., 2024, 2022) provided relevant data. The mean BMI was 25.2 ± 2.66 kg/m^2^, with variations observed across populations. Additionally, four studies (He et al., 2025; Ji et al., 2025; Wang et al., 2024; Liao et al., 2023) reported initial FMA for upper limb (FMA-UL) scores. The overall mean initial FMA-UL scores were 22.49 ± 13.67. The maximum possible score on the FMA-UL is 66, which indicates normal upper extremity motor function Song et al., (2019). Compared to the normal value, the reported scores are substantially lower, reflecting significant motor impairment among participants at baseline.

#### 3.2.2 Interventions

All included studies He et al., (2025); Zhang et al., (2025); Ji et al., (2025); Hou et al., (2024); Liu et al., (2023); Liao et al., (2023); Dun et al., (2023); Wang et al., (2024), (2022) implemented BCI-based intervention in the IG. The BCI systems integrated MI with different feedback modalities and assistive technologies. Specifically, seven studies Zhang et al., (2025); Hou et al., (2024); Liu et al., (2023); Liao et al., (2023); Dun et al., (2023); Wang et al., (2024), (2022) employed a BCI system integrating functional electrical stimulation with MI (BCI-FES-MI), while two studies He et al., (2025); Ji et al., (2025) utilized a BCI-robot-MI paradigm. Furthermore, although virtual feedback (VF) was the primary modality used across eight studies He et al., (2025); Zhang et al., (2025); Ji et al., (2025); Hou et al., (2024); Liu et al., (2023); Liao et al., (2023); Wang et al., (2024), (2022), mirror feedback (MF) was adopted in the study by Dun et al. (2023). All studies He et al., (2025); Zhang et al., (2025); Ji et al., (2025); Hou et al., (2024); Liu et al., (2023); Liao et al., (2023); Dun et al., (2023); Wang et al., (2024), (2022) provided conventional stroke rehabilitation programs in the CG, which were also administered concurrently to the IG. Notably, the study by He et al. (2025) was the only one to employ a placebo intervention alongside conventional rehabilitation. This intervention mimicked BCI therapy but was designed to have no genuine therapeutic effect.

The parameters of BCI-based intervention exhibited considerable heterogeneity across studies. Intensity was fixed in two trials He et al., (2025); Ji et al., (2025) and adjusted according to participant performance or tolerance in the remaining studies Zhang et al., (2025); Hou et al., (2024); Liu et al., (2023); Liao et al., (2023); Dun et al., (2023); Wang et al., (2024), (2022). It is important to highlight that in the included studies, BCI-Robot-MI typically operates at a fixed intensity, whereas BCI-FES-MI can be dynamically adjusted. Session frequency was uniformly set at one session per day, 5 days per week, with individual session duration ranging from 20 to 60 min, including 20 min He et al., (2025); Hou et al., (2024); Liu et al., (2023); Wang et al., (2022), 20–25 min Ji et al., (2025), 20–30 min Dun et al., (2023), 30 min Zhang et al., (2025); Wang et al., (2024), 45–60 min Liao et al., (2023). The total intervention duration varied from 2 weeks He et al., (2025) to 8 weeks Dun et al., (2023), though 4-week programs were most commonly implemented Ji et al., (2025); Hou et al., (2024); Wang et al., (2024), (2022). All interventions were conducted under professional supervision by therapists. This variability in BCI protocols reflects ongoing optimization of training parameters while maintaining methodological rigor through supervised implementation and consistent control conditions.

### 3.3 Methodological quality and risk of bias of included studies

The methodological quality and risk of bias of the included studies were evaluated using the PEDro scale and the Cochrane Risk of Bias tool. Table 3 presents the scores of the nine included RCTs based on the PEDro scale, with total scores ranging from 6 to 9. The study conducted by He et al. (2025) achieved the highest score of 9, thus classifying its methodological quality as “excellent.” Additionally, four studies (Zhang et al., 2025; Ji et al., 2025; Wang et al., 2024; Liu et al., 2023) obtained a quality score of 8 points, thereby designating these studies as “good” in methodological quality, while the other four studies (Hou et al., 2024; Liao et al., 2023; Dun et al., 2023; Wang et al., 2022), with PEDro scores of 6 or 7, were also deemed to be of good quality. The average PEDro score across all studies was 7.4, indicating good methodological quality.

**Table 3:** PEDro scale scores for the included studies.

**Study**	**Random allocation**	**Concealed allocation**	**Baseline similarity**	**Blind subjects**	**Blind therapists**	**Blind assessors**	**Adequate follow-up**	**Intention-to-treat analysis**	**Between-group comparisons**	**Point estimates and variability**	**Total score**
He et al. (2025)	Y	Y	Y	Y	N	Y	Y	Y	Y	Y	9
Zhang et al. (2025)	Y	Y	Y	N	N	Y	Y	Y	Y	Y	8
Ji et al. (2025)	Y	Y	Y	N	N	Y	Y	Y	Y	Y	8
Hou et al. (2024)	Y	N	Y	N	N	N	Y	Y	Y	Y	6
Wang et al. (2024)	Y	Y	Y	N	N	Y	Y	Y	Y	Y	8
Liu et al. (2023)	Y	Y	Y	N	N	Y	Y	Y	Y	Y	8
Liao et al. (2023)	Y	N	Y	N	N	Y	Y	Y	Y	Y	7
Dun et al. (2023)	Y	Y	Y	N	N	N	Y	Y	Y	Y	7
Wang et al. (2022)	Y	N	Y	N	N	N	Y	Y	Y	Y	6

PEDro, Physiotherapy Evidence Database; Y, Yes; N, No; The total score of PEDro: 10.

**Figure 2:**
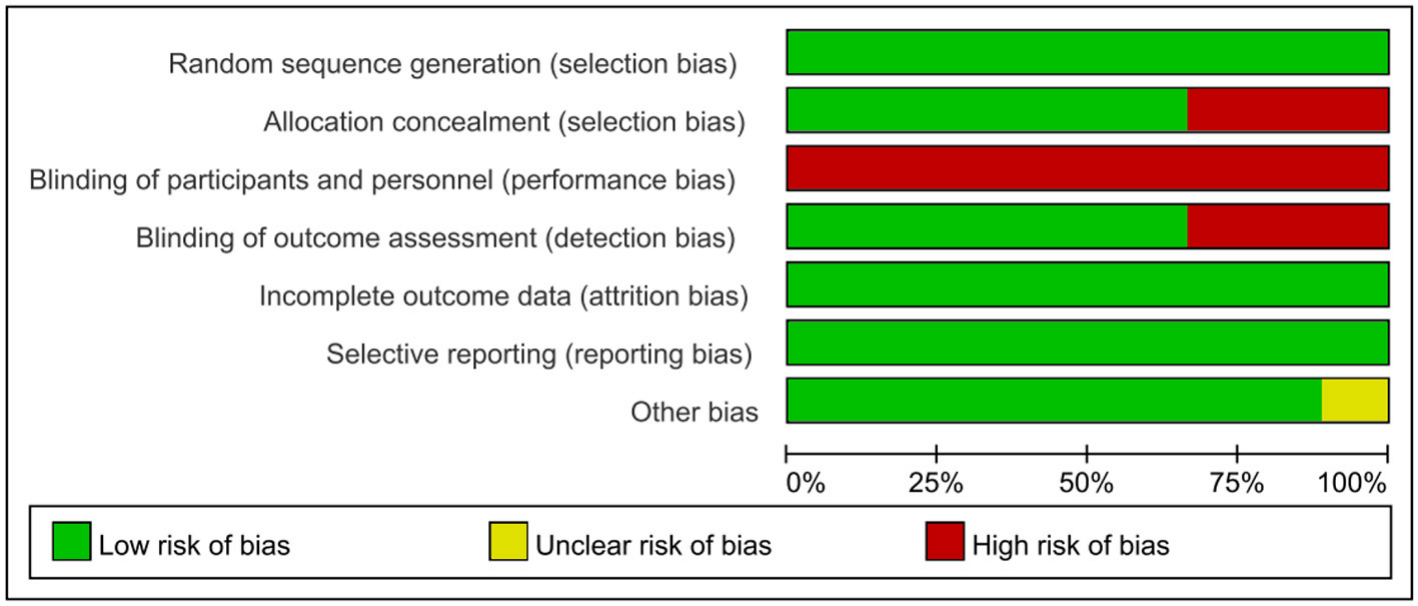
Risk of bias graph for included studies.

All included studies (He et al., 2025; Zhang et al., 2025; Ji et al., 2025; Hou et al., 2024; Liu et al., 2023; Liao et al., 2023; Dun et al., 2023; Wang et al., 2024, 2022) demonstrated key methodological strengths, including appropriate random allocation, baseline comparability, adequate follow-up, intention-to-treat analysis, between-group comparisons, alongside point estimates and variability. These elements collectively reduce selection, attrition and reporting biases, thus bolstering the credibility of the results. However, the risk of bias analysis presented in the risk of bias graph (Figure 2) highlights significant areas for concern. For instance, allocation concealment was missing in some studies (Hou et al., 2024; Liao et al., 2023; Wang et al., 2022), raising potential selection bias concerns. Regarding blinding procedures, six studies (He et al., 2025; Zhang et al., 2025; Ji et al., 2025; Wang et al., 2024; Liu et al., 2023; Liao et al., 2023) blinded outcome assessors, only one study (He et al., 2025) blinded subjects, and none blinded the therapists. This lack of comprehensive blinding poses a risk of performance and detection biases. Notably, performance bias should not be regarded as a type of preferential bias, as it frequently proves challenging or impractical to blind participants and therapists when executing intricate interventions (Rodrigues-Baroni et al., 2014). Therefore, although performance biases were present in these studies, this study acknowledged this variable and took it into account when interpreting the results.

### 3.4 Effects of interventions

#### 3.4.1 Effect of BCI-based rehabilitation on upper limb function

**Figure 3:**
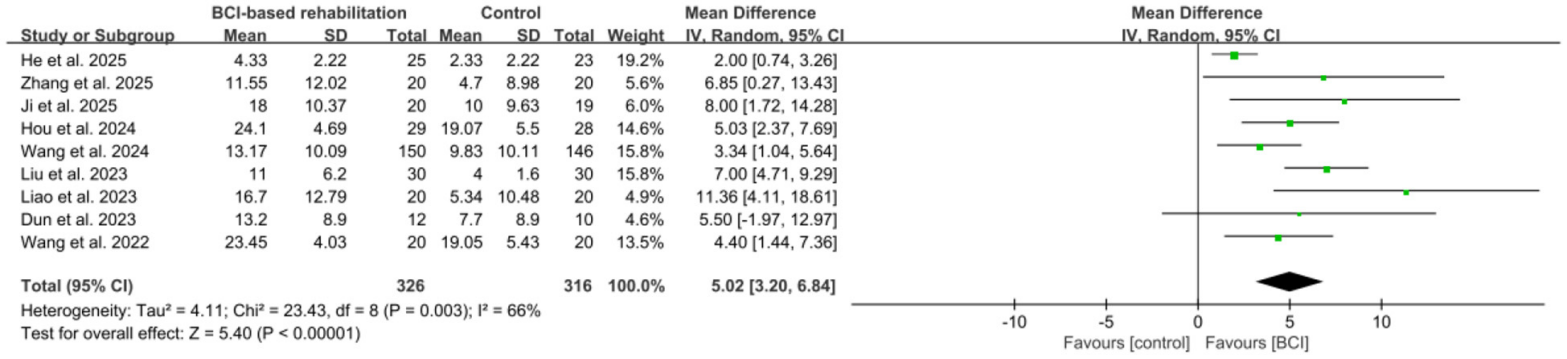
Forest plot for the pooled ES of BCI-based rehabilitation on upper limb function in early stroke patients compared to the CG.

All studies (He et al., 2025; Zhang et al., 2025; Ji et al., 2025; Hou et al., 2024; Liu et al., 2023; Liao et al., 2023; Dun et al., 2023; Wang et al., 2024, 2022) assessed upper limb function using the FMA scale. Five of these studies (He et al., 2025; Ji et al., 2025; Wang et al., 2024; Liu et al., 2023; Dun et al., 2023) reported outcome data as median and IQR. These data were converted to mean and SD for analysis in accordance with the pre-specified method. The aggregated data demonstrated that BCI-based rehabilitation significantly enhanced FMA scores for upper limb function in patients with early stroke compared to the CG (*n* = 642, MD = 5.02, 95% CI: 3.20, 6.84, *p* < 0.00001, *I*^2^ = 66%; Figure 3). This enhancement falls within the minimal clinically important difference (MCID) range for FMA in stroke populations, which is typically between 4.25 and 7.25 points according to distribution-based methods (Page et al., 2012). The sensitivity exclusion analysis revealed that no individual study notably affected the aggregated outcomes related to upper limb function. Additionally, to address potential selection bias from the predefined quality threshold, a sensitivity analysis was conducted by including three RCTs (Wu et al., 2020; Zhen et al., 2025; Tang and Zhang, 2021) that fulfilled other eligibility criteria but had been excluded solely because their PEDro score was below 6. The pooled result remained statistically significant and robust (*p* < 0.00001, Supplementary Figure S1), with the conclusion unchanged upon the sequential removal of each of these three lower-quality trials (Wu et al., 2020; Zhen et al., 2025; Tang and Zhang, 2021). The results of leave-one-out sensitivity analyses for upper limb function are presented in Supplementary Table S1.

Subgroup analyses were conducted for upper limb function based on study-level variables, including age (>60 years vs. ≤ 60 years), percentage female (>50 % vs. ≤ 50 %), time from onset ( ≤ 30 days vs. >30 days, corresponding broadly to the acute and subacute phases, respectively), BMI (>25 kg/m^2^ vs. ≤ 25 kg/m^2^), initial FMA-UL score (>23 vs. ≤ 23), feedback modalities (BCI-VF vs. BCI-MF), assistive technologies (BCI-robot vs. BCI-FES), time (≥30 min vs. < 30 min), duration (>3 weeks vs. ≤ 3 weeks). Subgroup analysis results for these indicators are shown in Table 4. Subgroup analyses revealed that BCI-based rehabilitation demonstrated significantly greater improvements in FMA-UE scores compared to the CG in subgroups defined by age, sex, time from onset, BMI, baseline FMA-UL score, intervention time, and duration (all *p* < 0.05). However, it is important to note that the subgroups for BMI >25 kg/m^2^ and initial FMA-UL score >23 each comprised data from only a single study. Findings from these subgroups should be interpreted with extreme caution, as they represent preliminary evidence. Notably, the magnitude of benefit on upper limb function did not differ significantly between the subgroups defined by time from onset ( ≤ 30 days vs. >30 days) (*p* = 0.24), indicating that the treatment effect is consistent in different early stages after stroke onset (acute vs. subacute phase). Similarly, no statistically significant between-subgroup differences were observed for the other patient or intervention characteristics (all *p* > 0.05), suggesting that treatment benefit may be consistent regardless of these patient or intervention characteristics. Regarding feedback modalities and assistive technologies, BCI-VF and BCI-FES demonstrated significant improvements in FMA-UE scores (all *p* < 0.00001), whereas no significant effects were observed for BCI-MF and BCI-robot (all *p* = 0.15). The comparison for the BCI-MF subgroup was based on a single study, and thus the non-significant finding lacks the power to draw any definitive conclusion. No statistically significant differences were found between these subgroup categories (all *p* > 0.05), meaning the overall treatment benefit may not be modified by this classification. The corresponding forest plots are presented in Supplementary Figures S2–S10.

**Table 4:** Results of subgroup analysis for upper limb function.

**Parameter**	**Subgroup**	**No. of studies**	**No. of patients**	**MD**	**95% CI**	**Effect *p*-value**	***I*^2^ (%)**	**Difference *p*-value**
Age (years)	>60	3	176	5.66	3.47, 7.86	< 0.00001	20	0.46
	≤ 60	5	466	4.41	1.90, 6.91	0.0006	74	
Female (%)	>50	2	97	7.26	1.33, 13.19	0.02	61	0.41
	≤ 50	7	545	4.63	2.61, 6.64	< 0.00001	67	
Time from onset (days)	>30	5	206	4.03	2.00, 6.07	0.0001	52	0.24
	≤ 30	4	436	5.02	3.20, 6.84	< 0.0001	61	
BMI (kg/m^2^)	>25	1	57	5.03	2.37, 7.69	0.0002	NA	0.55
	≤ 25	3	375	4.07	2.32, 5.81	< 0.00001	0	
Initial FMA-UL score	>23	1	296	3.34	1.04, 5.64	0.004	NA	0.37
	≤ 23	3	127	6.34	0.20, 12.48	0.04	78	
Feedback modalities	BCI-VF	8	620	5.03	3.11, 6.94	< 0.00001	70	0.90
	BCI-MF	1	22	5.50	−1.97, 12.97	0.15	NA	
Assistive technologies	BCI-robot	2	87	4.18	−1.48, 9.84	0.15	70	0.70
	BCI-FES	7	555	5.35	3.84, 6.86	< 0.00001	27	
Time (minutes)	≥30	3	376	6.21	1.50, 10.91	0.010	59	0.61
	< 30	6	266	4.87	2.61, 7.12	< 0.0001	73	
Duration (weeks)	>3	5	454	4.40	2.97, 5.83	< 0.00001	0	0.47
	≤ 3	4	188	6.00	1.95, 10.06	0.004	85	

BMI, Body mass index; FMA, Fugl-Meyer Assessment; UL, Upper limb; BCI, Brain-computer interface; FES, Functional electric stimulation; VF, Virtual feedback; MF, Mirror feedback; MD, Mean differences; CI, Confidence interval; NA, Not applicable.

#### 3.4.2 Effect of BCI-based rehabilitation on activities of daily living

**Figure 4:**
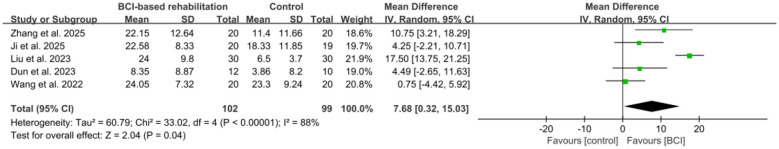
Forest plot for the pooled ES of BCI-based rehabilitation on activities of daily living in early stroke patients compared to the CG.

Five studies (Zhang et al., 2025; Ji et al., 2025; Liu et al., 2023; Dun et al., 2023; Wang et al., 2022) employed the MBI to evaluate activities of daily living. Among them, the studies by Ji et al. (2025) and Liu et al. (2023) provided medians and IQR, and their means and SD have been converted using the aforementioned method. The pooled data indicated that BCI-based rehabilitation was associated with an improvement in MBI scores for activities of daily living in early stroke patients relative to the CG (*n* = 201, MD = 7.68, 95% CI: 0.32, 15.03, *p* = 0.04; Figure 4). However, the statistical significance of this finding should be interpreted with great caution due to the very high heterogeneity (*I*^2^ = 88%; Figure 4)

and its borderline *p*-value. The magnitude of this pooled estimate exceeds the MCID for MBI in stroke, which is approximately 1.5–2.0 points based on the anchor-based method (Hsieh et al., 2007). Importantly, the sensitivity exclusion analysis showed that the statistical significance was lost following the sequential exclusion of studies by Zhang et al. (2025), Ji et al. (2025), and Dun et al. (2023), indicating limited robustness of the pooled result. Furthermore, a sensitivity analysis was performed by including the study by Tang and Zhang (2021), which met all other eligibility criteria but had been excluded solely due to a PEDro score below 6. The other two excluded lower-quality studies (Wu et al., 2020; Zhen et al., 2025) did not contribute data to the activities of daily living analysis. Including this trial resulted in a more statistically significant association (*p* = 0.002, Supplementary Figure S11), while removing it reduced the significance of the statistical association. This not only indicates that the effect estimate was not inflated by its inclusion but rather provides additional data supporting a potential beneficial effect of BCI-based rehabilitation on activities of daily living. However, it is important to note that the heterogeneity of the pooled result remained very high (*I*^2^ = 85%, Supplementary Figure S11), suggesting that caution is still warranted when interpreting this finding. The results of leave-one-out sensitivity analyses for activities of daily living are provided in Supplementary Table S2.

Subgroup analyses for activities of daily living were performed based on study-level variables with sufficient available data, including age (>60 years vs. ≤ 60 years), time from onset ( ≤ 30 days vs. >30 days), feedback modalities (BCI-VF vs. BCI-MF), assistive technologies (BCI-robot vs. BCI-FES), time (≥30 min vs. < 30 min), and duration (>3 weeks vs. ≤ 3 weeks). In contrast, subgroup analysis was not conducted for other variables, like percentage female (>50% vs. ≤ 50%), BMI (>25 kg/m^2^ vs. ≤ 25 kg/m^2^), and initial FMA-UL score (>23 vs. ≤ 23), due to fewer than three studies reporting outcomes related to activities of daily living. The results of the subgroup analysis about these indicators are presented in Table 5. Regarding patient characteristics, subgroup analysis showed that patients aged ≤ 60 years and with time from onset ≤ 30 days exhibited significantly greater improvement in activities of daily living (all *p* < 0.05), while no significant improvement was observed in patients aged >60 years and with time from onset >30 days (all *p* > 0.05). Remarkably, subgroup analysis for time from onset showed statistically significant differences between groups (*p* = 0.001), suggesting that different early stages after stroke onset (acute vs. subacute) may be an important effect modifier for activities of daily living, whereas subgroup analysis for age showed a borderline trend but did not reach statistical significance (*p* = 0.05). In terms of intervention characteristics, subgroup analysis demonstrated a statistically significant improvement in activities of daily living for interventions with time ≥30 min and duration ≤ 3 weeks (all *p* < 0.05), whereas no significant improvement was observed for time < 30 min and duration >3 weeks (all *p* > 0.05). Notably, the subgroup analysis for duration revealed statistically significant between-group differences (*p* = 0.001), indicating its potential role as an effect modifier, while no statistically significant difference was found for time (*p* = 0.52). Furthermore, subgroup comparisons based on feedback modalities (BCI-VF vs. BCI-MF) and assistive technologies (BCI-robot vs. BCI-FES) indicated no statistically significant effects on the improvement of activities of daily living (all *p* > 0.05), with no significant between-group differences observed (all *p* > 0.05). However, the results for the BCI-MF and BCI-robot subgroups, as well as for the intervention time ≥30 min subgroup, were each derived from only one study. Consequently, the observed effect in these particular subgroups should not be over-interpreted, and their true effects remain uncertain. The corresponding forest plots are presented in Supplementary Figures S12–S17.

**Table 5:** Results of subgroup analysis for activities of daily living.

**Parameter**	**Subgroup**	**No. of studies**	**No. of patients**	**MD**	**95% CI**	**Effect *p*-value**	***I*^2^ (%)**	**Difference p-value**
Age (years)	>60	2	79	2.12	−1.92, 6.15	0.30	0	0.05
	≤ 60	3	201	11.35	3.20, 19.50	0.006	82	
Time from onset (days)	>30	3	101	2.69	−0.82, 6.20	0.13	0	0.001
	≤ 30	2	100	14.95	8.53, 21.36	< 0.00001	60	
Feedback modalities	BCI-VF	4	179	8.41	−0.36, 17.17	0.06	90	0.50
	BCI-MF	1	22	4.49	−2.65, 11.63	0.22	NA	
Assistive technologies	BCI-robot	1	39	4.25	−2.21, 10.71	0.20	NA	0.45
	BCI-FES	4	162	8.49	−0.35, 17.34	0.06	90	
Time (minutes)	≥30	1	40	10.75	3.21, 18.29	0.005	NA	0.52
	< 30	4	161	6.93	−2.04, 15.91	0.13	91	
Duration (weeks)	>3	3	101	2.69	−0.82, 6.20	0.13	0	0.001
	≤ 3	2	100	14.95	8.53, 21.36	< 0.00001	60	

BMI, Body mass index; FMA, Fugl-Meyer Assessment; UL, Upper limb; BCI, Brain-computer interface; FES, Functional electric stimulation; VF, Virtual feedback; MF, Mirror feedback; MD, Mean differences; CI, Confidence interval; NA, Not applicable.

#### 3.4.3 Effect of BCI-based rehabilitation on adverse events

Data on adverse events were available from a single included study (Wang et al., 2024). Given that only one study reported on adverse events, a meta-analysis was not feasible for this outcome. Thus, the results are presented descriptively. The results from this study indicated that adverse outcomes occurred in 33 patients (22.00%) in the IG and 31 patients (21.23%) in the CG. Statistical analysis showed no significant difference in the rates of adverse events between the two groups (*p* = 0.87). Furthermore, serious adverse outcomes were reported in six patients (4.00%) in the IG and three patients (2.05%) in the CG, with no significant difference between groups (*p* = 0.50). Adverse events and serious adverse events were classified according to primary system organ class and preferred term, like nervous system disorders, general disorders and mortality. Based on the available data from this single study, BCI-based rehabilitation was not associated with a significantly increased rate of adverse events compared to conventional rehabilitation. However, the evidence on safety is insufficient to draw firm conclusions due to the lack of data from other trials.

### 3.5 Quality of evidence for results

Grading of the evidence using the GRADE approach yielded moderate certainty for upper limb function, low for daily living activities, and very low for adverse events. For upper limb function, the quality of evidence was downgraded due to substantial statistical heterogeneity (*I*^2^ = 66%), although all included studies (He et al., 2025; Zhang et al., 2025; Ji et al., 2025; Hou et al., 2024; Liu et al., 2023; Liao et al., 2023; Dun et al., 2023; Wang et al., 2024, 2022) demonstrated a low risk of bias, with PEDro scores of 6 or higher. No issues of indirectness or imprecision were detected, as the effect was precise with an MD of 5.02 points (95% CI: 3.20–6.84) within the MCID range. Regarding activities of daily living, the risk of bias was judged to be low, as all five included studies (Zhang et al., 2025; Ji et al., 2025; Liu et al., 2023; Dun et al., 2023; Wang et al., 2022) achieved PEDro scores of 6 or higher, and no indirectness was identified. However, the evidence was downgraded owing to very serious inconsistency (*I*^2^ = 88%), additional concerns about imprecision (wide 95% CI: 0.32–15.03), and observed subgroup differences based on time from onset and intervention duration, suggesting effect modification. Additionally, data on adverse events were available from only one study (Wang et al., 2024). The evidence was severely downgraded, despite a non-serious risk of bias (PEDro scores ≥6) and no detected indirectness. This decision was driven by the inability to assess inconsistency, compounded by very serious imprecision. The sparse data provide an insufficient sample size, precluding a reliable estimate of adverse event incidence and limiting the ability to rule out a clinically important risk difference. Given the insufficient number of eligible studies (*n* = 9), the statistical power to reliably detect publication bias was low, thus precluding a formal assessment for outcome indicators (Cumpston et al., 2019). The GRADE evaluation for outcomes is illustrated in Supplementary Table S3.

## 4 Discussion

This study assesses the available evidence for the effects of BCI-based rehabilitation on upper limb function, activities of daily living, and adverse events in patients with early stroke. It provides consistent evidence that BCI-based rehabilitation significantly improves primary upper limb function and suggests a potential benefit for secondary activities of daily living in patients who have experienced a stroke in the early stages. However, the conclusion regarding activities of daily living must be interpreted with caution due to very high statistical heterogeneity and the loss of statistical significance in sensitivity analyses, indicating that the pooled estimate is not robust and the certainty of evidence is low. These results tentatively suggest that BCI-based rehabilitation may serve as a promising therapeutic strategy for improving functional recovery in early stroke patients. However, evidence regarding adverse events remains extremely limited, as it is derived from only one included study. While this single trial reported no significant increase in risk compared to conventional rehabilitation, the current evidence base is insufficient to draw any definitive conclusions about the safety profile of BCI-based rehabilitation. Thus, further large-scale RCTs are needed to comprehensively evaluate the safety and wider effectiveness of BCI-based rehabilitation for upper limb dysfunction in early stroke populations. Future trials should also employ standardized definitions and reporting frameworks for adverse events to facilitate evidence synthesis.

Subgroup analyses indicated that there were no statistically significant differences in the enhancement of upper limb function across diverse patient demographics and intervention attributes, implying that BCI-based rehabilitation might possess extensive applicability. In contrast, for activities of daily living, greater benefits were observed in patients within 30 days after stroke onset and with intervention durations of no more than 3 weeks, indicating that time after stroke onset and duration of intervention may be important effect modifiers. This may be attributable to enhanced neural plasticity in the early phase following stroke and the appropriate intervention dosage within a critical time window. Although BCI-VF and BCI-FES showed significant improvements in upper limb function, no statistically significant differences were found between subgroups, implying that these technical variations may not affect the overall treatment benefit. Given the scarcity of studies available for certain subgroup comparisons, particularly with only one or two instances, further RCTs are warranted to validate these findings and explore other potential moderating factors deeply, such as feedback modalities and assistive technologies.

This systematic review and meta-analysis demonstrated that BCI-based rehabilitation significantly improved upper limb function in patients with early stroke, with the magnitude of improvement falling within the MCID of the FMA, indicating clinical relevance of the intervention. This finding is consistent with previous systematic reviews (Lo et al., 2024; Li et al., 2025; Xie et al., 2022; Ren et al., 2024; Baniqued et al., 2021; Carvalho et al., 2019; Peng et al., 2022; Yang et al., 2021; Shou et al., 2023; Nojima et al., 2022), although those studies did not specifically analyze early stroke patients separately, instead including mixed cohorts of early and chronic stroke populations. The present study strengthens the evidence by focusing specifically on early stroke patients. This temporal specificity may allow for a more precise evaluation of intervention effects during the critical period of enhanced neuroplasticity following stroke onset (Patel et al., 2019; Hebert et al., 2016). The observed functional improvements may stem from the fact that BCI-based rehabilitation promotes multi-level neurophysiological remodeling through a closed-loop feedback mechanism (Sebastián-Romagosa et al., 2020; Li et al., 2025). This system effectively promotes functional reorganization of the sensorimotor cortex and significantly enhances sensorimotor integration capabilities by decoding neural signals related to motor intention in real time and providing precise feedback (Li et al., 2025; Ren et al., 2024). The process drives neural plasticity by strengthening neural pathways involved in motor planning and execution, promoting functional optimization of the corticospinal tract (Sebastián-Romagosa et al., 2020; Li et al., 2025; Xie et al., 2022). The mechanism is particularly important in the early stages of stroke, as the brain is in a critical window of neural plasticity at this time (Zhang et al., 2024; Patel et al., 2019). Introducing BCI-based rehabilitation at this time can maximize the activation of the neural coupling between motor imagery and actual execution, and further accelerate functional recovery by optimizing neural circuit reorganization (Zanona et al., 2023; Wu et al., 2020). Furthermore, integrating visual or multimodal feedback modes can not only enhance training engagement, but also promote the depth and persistence of motor learning and adaptive neural remodeling through multisensory synergistic stimulation (Xie et al., 2022; Zhang et al., 2025). Subgroup analysis showed that BCI-based rehabilitation demonstrated stable efficacy across different population characteristics (including patients in different early stages after stroke, such as acute and subacute phases) and intervention protocols, suggesting that this therapy may have broad applicability in early stroke patients. It is worth noting that while interventions using BCI-VF and BCI-FES demonstrated significant improvements, BCI-MF and BCI-robot configurations did not show statistically significant benefits. This observation may be attributed to limited subgroup sample sizes, variations in implementation protocols, or inadequate statistical power (Lo et al., 2024; Li et al., 2025). However, no significant differences were observed in direct comparisons between subgroups, suggesting that while specific technical configurations may modulate the magnitude of therapeutic effects to some extent, the overall treatment benefit remains robust across different implementation approaches. Although these results are encouraging, the significant heterogeneity and limited number of studies focusing on specific subgroup comparisons underscore the need for caution in interpreting the findings. Future RCTs should prioritize optimizing intervention parameters, including feedback types and technical implementation, while adopting standardized outcome measures to further validate effects of BCI-based rehabilitation across different intervention modes and explore its differential impacts in early vs. chronic stroke patients.

This study found that BCI-based rehabilitation was associated with an enhancement in daily living activities in patients with early stroke, with the point estimate exceeding the MCID threshold, suggesting potential clinical relevance. This result is directionally consistent with some previous research findings (Li et al., 2025; Xie et al., 2022; Peng et al., 2022; Shou et al., 2023), which tentatively support the positive role of BCI-based rehabilitation in improving post-stroke daily function, although those studies predominantly included mixed cohorts of both early and chronic stroke patients. The potential improvement in activities of daily living likely stems from BCI-based rehabilitation effectively enhancing sensorimotor integration and cortico-spinal pathway function through real-time decoding of neural signals associated with motor intention and providing multimodal closed-loop feedback, thereby promoting task-specific neuroplasticity and motor learning (Lo et al., 2024; Xie et al., 2022). Moreover, it may also involve both the direct impact of BCI-based rehabilitation on upper limb functional recovery and indirect benefits on cognitive and perceptual processes, which collectively may contribute to the transfer of acquired skills to practical daily activities through adaptive reorganization of neural circuits and enhanced motor control efficiency (Xie et al., 2022; Peng et al., 2022). However, it is crucial to interpret this finding with caution. Pooled analyses revealed substantial heterogeneity in the outcomes, and sensitivity analyses indicated that the conclusions were susceptible to individual studies, reflecting an unstable evidence base of low certainty. This variability likely arises from variations across studies in patient baseline characteristics, intervention parameters, or BCI technical protocols. Subgroup analyses further demonstrated that patients who initiated intervention within 30 days post-stroke and received a treatment course of no more than 3 weeks exhibited more significant improvements in activities of daily living, suggesting that early intervention and an optimized treatment duration may represent critical factors for enhancing functional outcomes. This may indicate that the integration of regained motor capacity into complex, daily tasks is more readily enhanced when BCI-based rehabilitation is applied during the acute phase of heightened spontaneous recovery and cortical excitability (Chen et al., 2026; Qiao et al., 2023). Conversely, in the >30 days (subacute-phase) subgroup, the lack of a significant effect on activities of daily living could suggest that altering established daily routines or compensation strategies requires different or more prolonged intervention (Chen et al., 2026; Qiao et al., 2023). The underlying mechanism may be that there is temporal heterogeneity in the early post-stroke window of neuroplasticity and functional remodeling, and the enhancing effect is not consistent within 3 months (Patel et al., 2019). Implementing intensive and targeted BCI-based rehabilitation within the acute window could maximize neural adaptive benefits while mitigating potential fatigue or reduced adaptability associated with extended training periods (Patel et al., 2019; Zhang et al., 2025; Ji et al., 2025). Remarkably, certain subgroups, such as older patients and those receiving extended intervention periods, did not demonstrate comparable improvements in efficacy, highlighting the importance of individual differences in rehabilitation responsiveness. Therefore, although this study primarily analyzed the efficacy of BCI-based rehabilitation within 3 months post-stroke, the subgroup results highlight the potential importance of intervention timing and encourage future trials to clearly stratify participants according to clinically defined acute and subacute phases to optimize intervention protocols. Overall, although BCI-based rehabilitation shows a signal of potential benefit for improving activities of daily living in early-stage stroke patients, the current evidence is not robust. Future rigorously designed RCTs with well-characterized patient populations remain imperative to systematically evaluate efficacy variations across different BCI modalities, dose-response relationships, and their impacts on long-term functional independence, thus establishing an evidence-based foundation for developing individualized and precision rehabilitation strategies.

This systematic review evaluated the available evidence on the safety profile of BCI-based rehabilitation in patients with early stroke. Data from the single included study that reported on this outcome showed no significant difference in the risk of adverse events between groups (Wang et al., 2024), which is consistent with the conclusions of the only meta-analysis currently focusing on the safety of BCI-based rehabilitation in stroke (Xie et al., 2022). The relatively good safety profile suggested by this limited data may be attributed to the non-invasive nature of BCI, the relatively stable physiological state of early stroke patients, structured monitoring protocols during training, and the effective integration with traditional rehabilitation programs, which collectively help minimize potential risks (Xie et al., 2022; Wang et al., 2024). However, it must be emphatically acknowledged that the current safety evidence is severely limited, being heavily reliant on findings from a single study (Wang et al., 2024). The inability to perform a meta-analysis precludes any definitive conclusions regarding safety, and the generalizability of these preliminary findings is substantially constrained. Furthermore, previous systematic reviews (Lo et al., 2024; Li et al., 2025; Xie et al., 2022; Ren et al., 2024; Baniqued et al., 2021; Carvalho et al., 2019; Peng et al., 2022; Yang et al., 2021; Shou et al., 2023; Nojima et al., 2022) have often either omitted adverse event data or based their analyses on mixed stroke populations, potentially obscuring risk profiles specific to early-phase patients. This also reflects that most studies prioritize efficacy outcomes, while systematic safety monitoring and reporting mechanisms remain underdeveloped, representing a critical gap in the existing literature. Importantly, definitions and methods for reporting adverse events likely vary, hindering comparability and synthesis. Future research should strengthen the systematic collection and standardized classification of adverse events in RCTs, such as using the major system organ classification standard, and adopt consistent reporting frameworks to facilitate evidence synthesis. More representative safety studies across different stroke phases are needed to comprehensively evaluate the risk–benefit profile of BCI-based rehabilitation, thereby establishing a reliable evidence base for its clinical translation.

Current evidence suggests that BCI-based rehabilitation may be a promising intervention to improve functional recovery in patients with early-stage stroke. Given its role in promoting functional recovery during the critical window of neuroplasticity, early implementation of BCI-based rehabilitation is recommended to optimize functional outcomes. However, clinical translation requires cautious interpretation due to several limitations and substantial heterogeneity in the current evidence. Firstly, the number of high-quality RCTs in this field is limited, with only nine studies ultimately included, resulting in an insufficient total sample size. This could introduce small sample bias and limit the generalizability and robustness of the conclusions. Secondly, a potential source of bias may be related to the methodological quality thresholds employed. To enhance the internal validity of the evidence synthesis, a pre-specified methodological quality threshold (PEDro score ≥6) was applied for the primary analyses. This decision aimed to prioritize the integration of trial evidence with lower risk of bias and greater methodological rigor, thereby providing a more reliable basis for evaluating the efficacy of BCI-based rehabilitation in this emerging field. However, this criterion itself may introduce limitations, particularly selection bias. Since smaller trials or those with negative results are sometimes associated with lower reporting quality and may be disproportionately excluded, which could potentially overestimate the pooled effect size and limit the comprehensiveness of the evidence base as well as the generalizability of the conclusions. To directly test this possibility and address this concern, a sensitivity analysis was conducted that included trials which had been excluded due to a PEDro score below 6 but met all other eligibility criteria. For upper limb function, the beneficial effect remained statistically significant and clinically meaningful after incorporating lower-quality trials, confirming the robustness of this primary conclusion. For activities of daily living, including an additional lower-quality trial increased the statistical significance of the pooled estimate but did not resolve the issue of high heterogeneity or the fragility of the result observed in leave-one-out analyses. Thus, while the application of a quality threshold requires careful consideration, it is demonstrated by sensitivity analyses that the key finding regarding upper limb function is robust, and that the more tentative finding for activities of daily living is not solely an artifact of the inclusion criteria. Future clinical trials in this field should further improve the rigor of methodological design and the completeness of reporting to fundamentally reduce the limitations arising from such methodological trade-offs. Besides, significant heterogeneity was observed in both upper limb function and daily living activity outcomes, suggesting variations in patient baseline characteristics or intervention protocols across studies. Although subgroup and sensitivity analyses were conducted, residual heterogeneity complicates the interpretation of the results. Moreover, due to the limited number of included studies, it was not possible to conduct meta-regression analysis to explore the relationship between effect size and baseline covariates such as sex ratio and mean age, nor was it possible to conduct a formal statistical assessment of publication bias. Therefore, the interpretation of results needs to take into account the potential risk of bias. Additionally, several subgroup analyses were based on only a single included study, which limits the robustness of the findings. This pertains to specific subgroup analyses for both upper limb function (e.g., BMI >25 kg/m^2^, baseline FMA-UL score >23, and the BCI-MF feedback modality) and activities of daily living (e.g., the BCI-MF feedback modality, the BCI-robot assistive technology, and the intervention time ≥30 min). While presented for completeness and to generate hypotheses, the estimates from these single-study subgroups are unstable and lack generalizability. They should be considered exploratory findings that require validation in future, adequately powered studies. It is important to note that considerable variability in BCI-based rehabilitation parameters across studies further limits the ability to derive unified clinical recommendations.

Beyond these limitations, a key methodological consideration that limits the precise interpretation of our findings is that, in nearly all included trials, BCI-based rehabilitation was administered as an adjunct to conventional therapy. While this pragmatic design reflects real-world clinical practice of integrating novel therapies into standard treatment, it presents significant challenges in isolating incremental effects specifically attributable to BCI-based rehabilitation. The benefits observed in the IG may stem from the BCI-based rehabilitation intervention itself, as well as the increase in total treatment time or improved patient engagement compared to the CG. This overlapping effect makes it difficult to discern whether improvements are driven by neuroplasticity mechanisms unique to BCI-based rehabilitation or are simply due to additional practice. The study by He et al. (2025) employed a sham BCI-based rehabilitation control in addition to conventional therapy in the CG, which is a noteworthy exception and helps alleviate this concern by controlling for the non-specific effects of additional treatment time and technological interaction. Consequently, their positive findings provide stronger evidence for the specific efficacy of BCI-based rehabilitation. However, for the majority of studies, the pooled estimates in this study likely reflect the value-added effect of BCI-based rehabilitation combined with conventional rehabilitation, rather than the effect of BCI-based rehabilitation alone. Future trials should consider employing active placebo or dose-matched control designs to better isolate the specific therapeutic component of BCI-based interventions. Therefore, when interpreting the current results, it should be recognized that these results support BCI-based rehabilitation combined with conventional rehabilitation, but the specific role of BCI-based rehabilitation itself still needs to be further clarified through more refined comparative designs. Overall, the certainty of evidence is limited by statistical heterogeneity, imprecision, and data sparsity. The grading of outcome evidence was downgraded due to considerable heterogeneity and wide confidence intervals. Furthermore, the limited number of studies substantially constrains the reliability of the evidence, with only one trial providing complete safety data, thereby compromising the overall evidence quality and resulting in very low certainty for safety outcomes, which precludes a robust assessment of long-term safety. Finally, the short follow-up durations in most trials also limit evaluation of the long-term sustainability of BCI-based rehabilitation effects. Future research should employ sham-controlled RCT designs and conduct large-scale, methodologically rigorous clinical trials to establish optimized BCI-based rehabilitation protocols, systematically evaluate the efficacy of different configurations and dose-response relationships, incorporate standardized safety monitoring, and investigate long-term functional outcomes across diverse stroke populations.

## 5 Conclusions

This systematic review and meta-analysis provide robust evidence that BCI-based rehabilitation significantly improves primary upper limb function in early stroke patients. Improvement in upper limb function is generally consistent across different demographic characteristics and intervention parameters. Furthermore, BCI-based rehabilitation is associated with a potential benefit for secondary activities of daily living, particularly in patients within 30 days of stroke onset and those receiving interventions of shorter duration ( ≤ 3 weeks). However, this finding for activities of daily living must be interpreted with considerable caution due to substantial heterogeneity, limited robustness in sensitivity analyses, and consequently low certainty of the evidence. Regarding safety, preliminary data from a single study suggest no significant difference in adverse event rates, but the evidence base is currently insufficient to draw definitive conclusions about the safety profile of BCI-based rehabilitation in this population. More well-designed multi-center, randomized, double-blind, placebo-controlled trials are required to confirm the potential benefit on activities of daily living with more robust evidence, and to evaluate the long-term efficacy and safety of optimized BCI-based rehabilitation protocols for early stroke patients, including feedback modalities, assistive technologies, and intervention dosages.

## Data Availability

The original contributions presented in the study are included in the article/Supplementary material, further inquiries can be directed to the corresponding authors.
