# Supplementary material for: Effects of brain-computer interface-based rehabilitation on upper limb function, activities of daily living, and adverse events in patients with early stroke: a systematic review and meta-analysis
Source: Front Aging Neurosci. 2026 Mar 24;18:1737740. doi: 10.3389/fnagi.2026.1737740 (PMC13055532; doi:10.3389/fnagi.2026.1737740)
Supplement: Supplementary file 1 [file Data_Sheet_1.doc]

**Supplementary Material**s

| **Table S1 Leave-one-out sensitivity analysis for upper limb function** | | | |
| --- | --- | --- | --- |
| **Study removed** | **Pooled ES (95% CI)** | **I²** **(%)** | **P value** |
| He et al. 2025 | 5.46 [4.03, 6.89] | 21 | 0.26 |
| Zhang et al. 2025 | 4.93 [3.02, 6.83] | 69 | 0.002 |
| Ji et al. 2025 | 4.82 [2.95, 6.69] | 68 | 0.003 |
| Hou et al. 2024 | 5.11 [3.01, 7.21] | 69 | 0.002 |
| Wang et al. 2024 | 5.46 [3.27, 7.66] | 70 | 0.002 |
| Liu et al. 2023 | 4.42 [2.69, 6.16] | 52 | 0.04 |
| Liao et al. 2023 | 4.64 [2.90, 6.38] | 63 | 0.008 |
| Dun et al. 2023 | 5.03 [3.11, 6.94] | 70 | 0.002 |
| Wang et al. 2022 | 5.21 [3.11, 7.31] | 70 | 0.002 |
| **PEDro score PEDro < 6** |  |  |  |
| Zhen et al. 2025 | 5.40 [3.62, 7.17] | 78 | <0.00001 |
| Tang and Zhang 2021 | 5.22 [3.36, 7.08] | 81 | <0.00001 |
| Wu et al. 2020 | 4.29 [2.95, 5.63] | 63 | <0.00001 |
| ES=Effect Size, CI=Confidence interval | | | |

| **Table S2 Leave-one-out sensitivity analysis for activities of daily living** | | | |
| --- | --- | --- | --- |
| **Study removed** | **Pooled ES (95% CI)** | **I²** **(%)** | **P value** |
| Zhang et al. 2025 | 6.93 [-2.04, 15.91] | 91 | <0.00001 |
| Ji et al. 2025 | 8.49 [-0.35, 17.34] | 90 | <0.00001 |
| Liu et al. 2023 | 4.48 [0.46, 8.49] | 35 | 0.20 |
| Dun et al. 2023 | 8.41 [-0.36, 17.17] | 90 | <0.00001 |
| Wang et al. 2022 | 9.56 [2.17, 16.95] | 83 | 0.0004 |
| **PEDro score PEDro < 6** |  |  |  |
| Tang and Zhang 2021 | 7.68 [0.32, 15.03] | 88 | 0.04 |
| ES=Effect Size, CI=Confidence interval | | | |

| **Table S3 GRADE assessment for outcomes** | | | | | | |
| --- | --- | --- | --- | --- | --- | --- |
| **Outcome indicators** | **Serious risk of bias** | **Inconsistency** | **Indirectness** | **Imprecision** | **Publication bias** | **Quality of evidence** |
| FMA | N | Y | N | N | U | Moderate |
| MBI | N | Y | N | Y | U | Low |
| Adverse events | N | NA | N | Y | U | Very Low |
| GRADE=Grading of Recommendations Assessment, Development and Evaluation, FMA=Fugl-Meyer Assessment, MBI=Modified Barthel Index, Y=Yes, N=No, NA=Not applicable, U= Undetected | | | | | | |


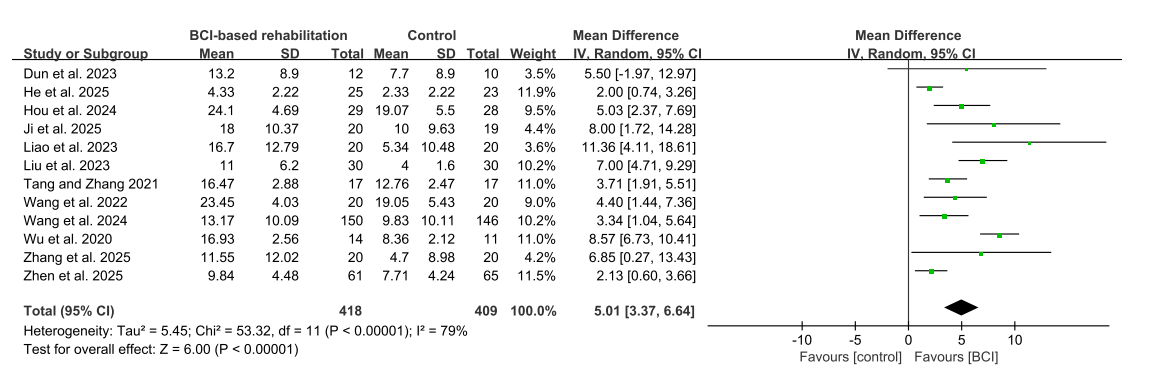


Figure S1 Forest plot for the pooled ES of BCI-based rehabilitation on upper limb function from three additional low-quality studies


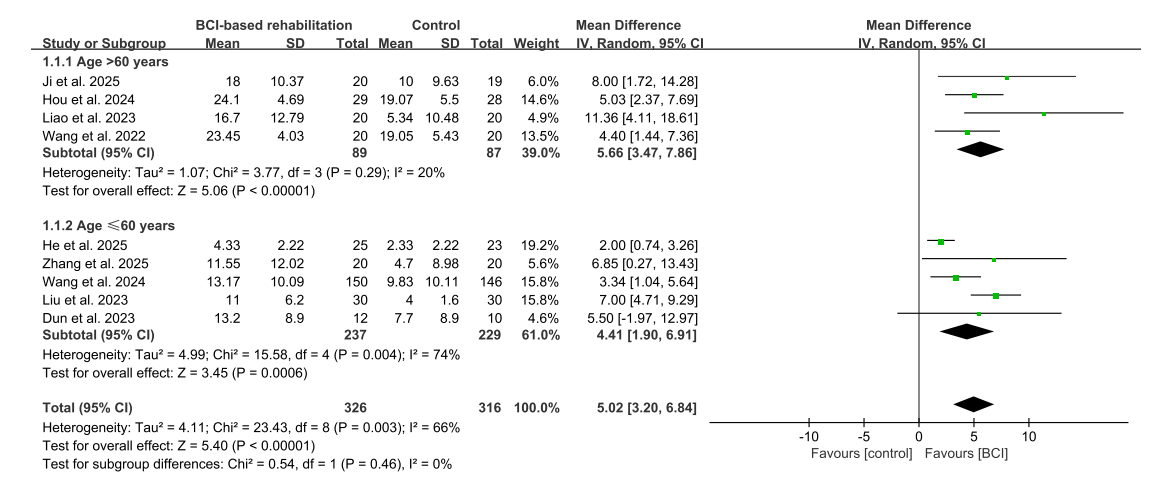


Figure S2 Forest plot for the pooled ES of BCI-based rehabilitation on upper limb function in age subgroup analysis


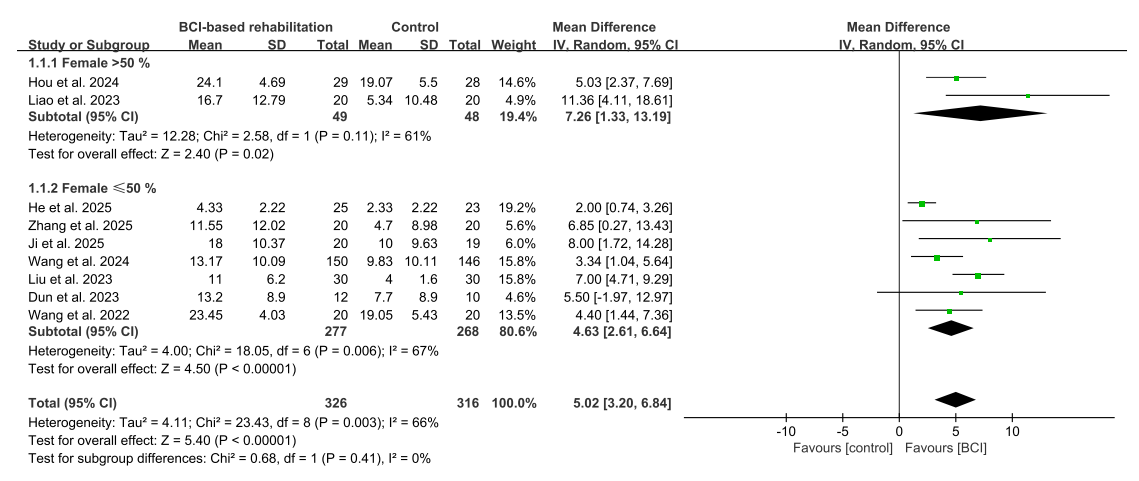


Figure S3 Forest plot for the pooled ES of BCI-based rehabilitation on upper limb function in % female subgroup analysis


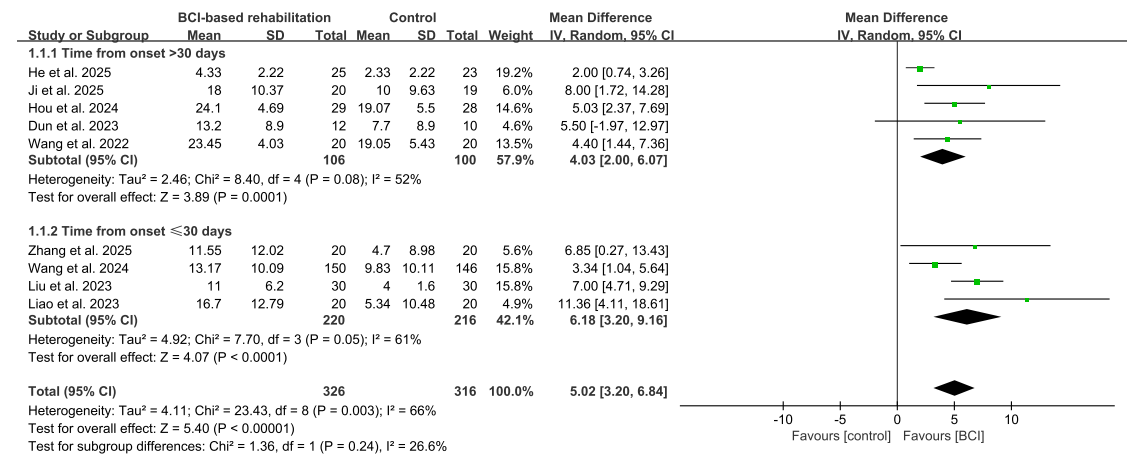


Figure S4 Forest plot for the pooled ES of BCI-based rehabilitation on upper limb function in time from onset subgroup analysis


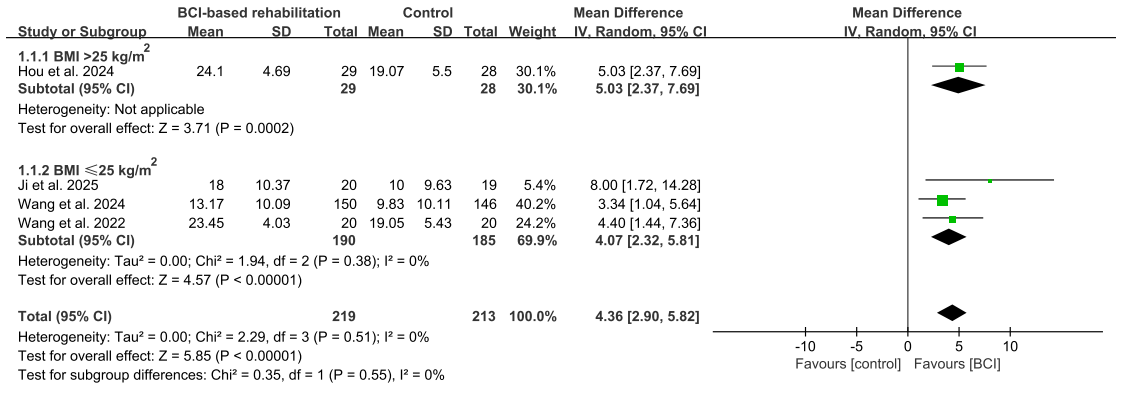


Figure S5 Forest plot for the pooled ES of BCI-based rehabilitation on upper limb function in body mass index subgroup analysis


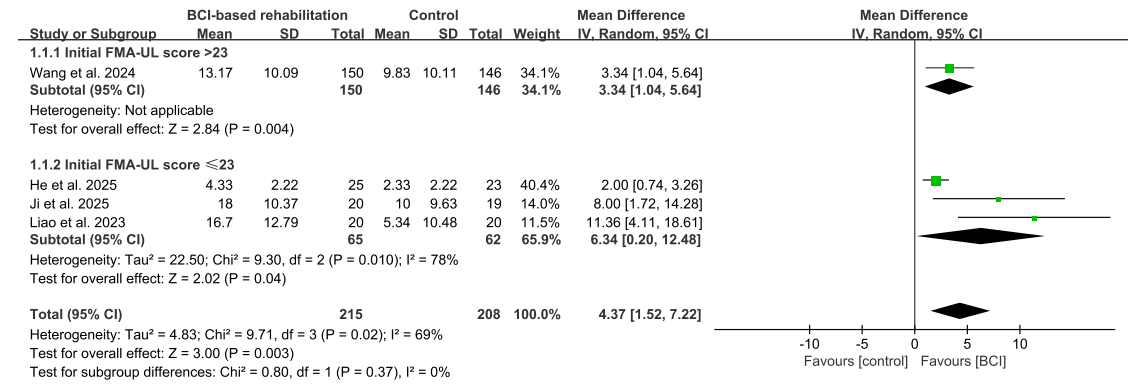


Figure S6 Forest plot for the pooled ES of BCI-based rehabilitation on upper limb function in initial FMA-UL score subgroup analysis


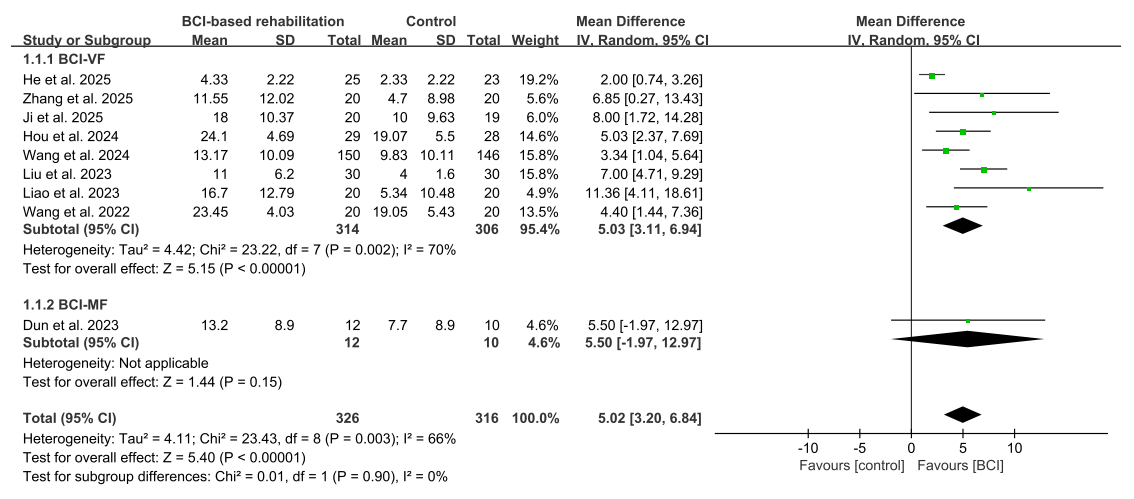


Figure S7 Forest plot for the pooled ES of BCI-based rehabilitation on upper limb function in feedback modalities subgroup analysis


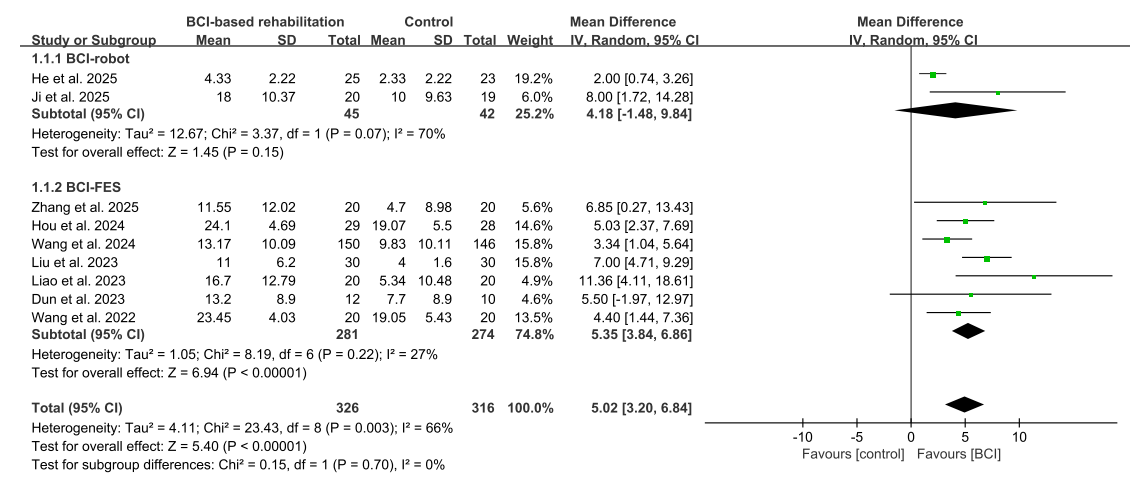


Figure S8 Forest plot for the pooled ES of BCI-based rehabilitation on upper limb function in assistive technologies subgroup analysis


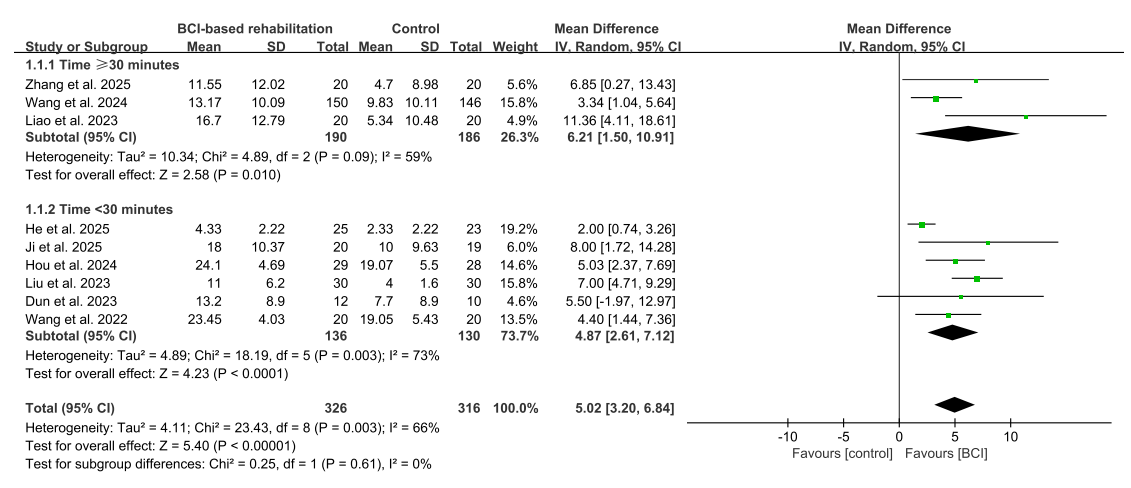


Figure S9 Forest plot for the pooled ES of BCI-based rehabilitation on upper limb function in time subgroup analysis


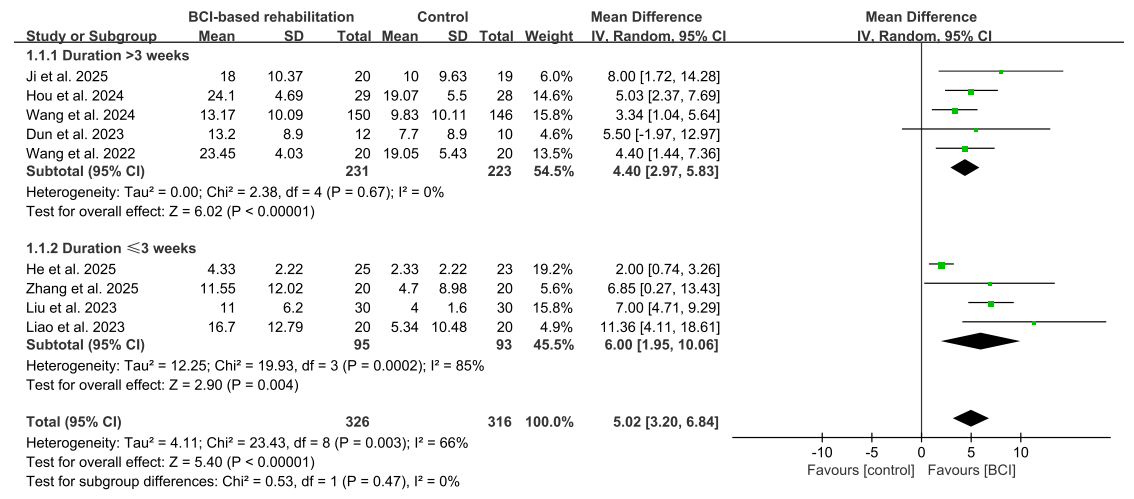


Figure S10 Forest plot for the pooled ES of BCI-based rehabilitation on upper limb function in duration subgroup analysis


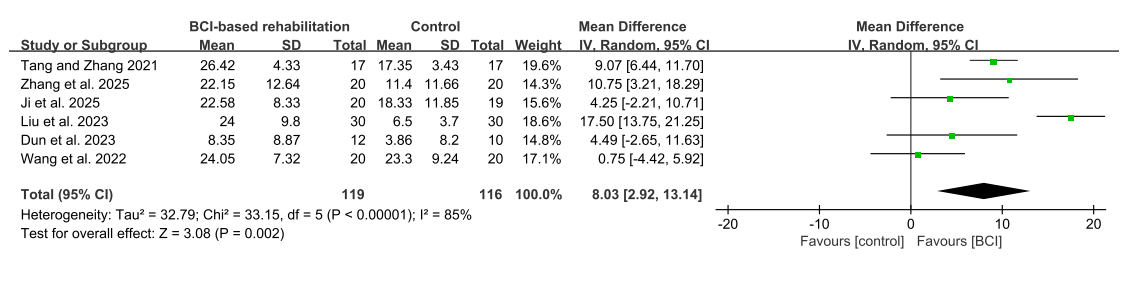


Figure S11 Forest plot for the pooled ES of BCI-based rehabilitation on activities of daily living from one additional low-quality study


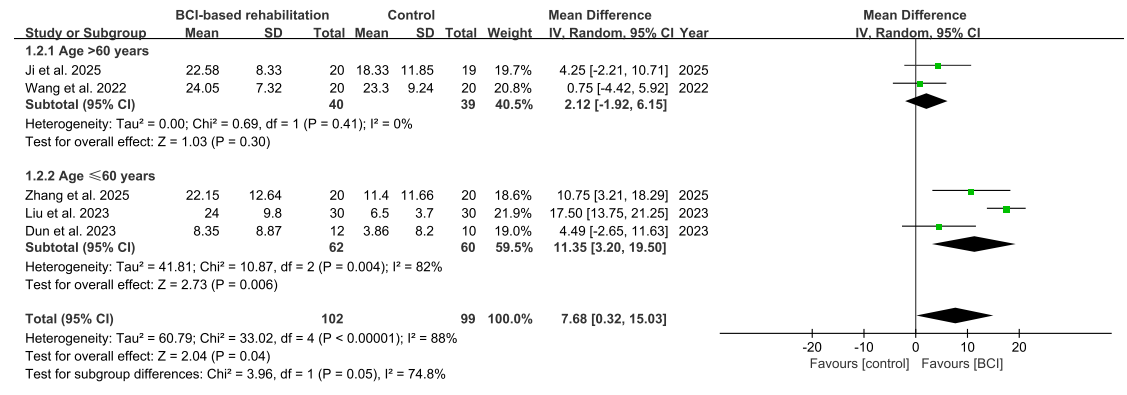


Figure S12 Forest plot for the pooled ES of BCI-based rehabilitation on activities of daily living in age subgroup analysis


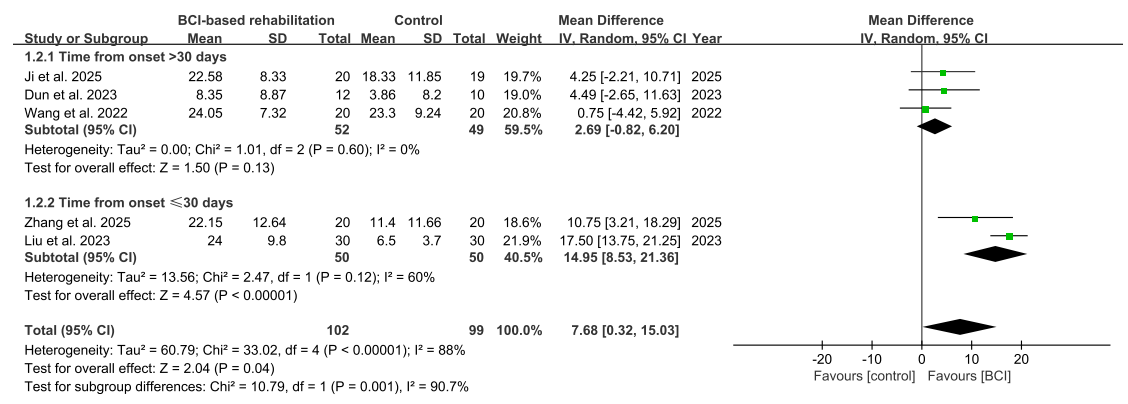


Figure S13 Forest plot for the pooled ES of BCI-based rehabilitation on activities of daily living in time from onset subgroup analysis


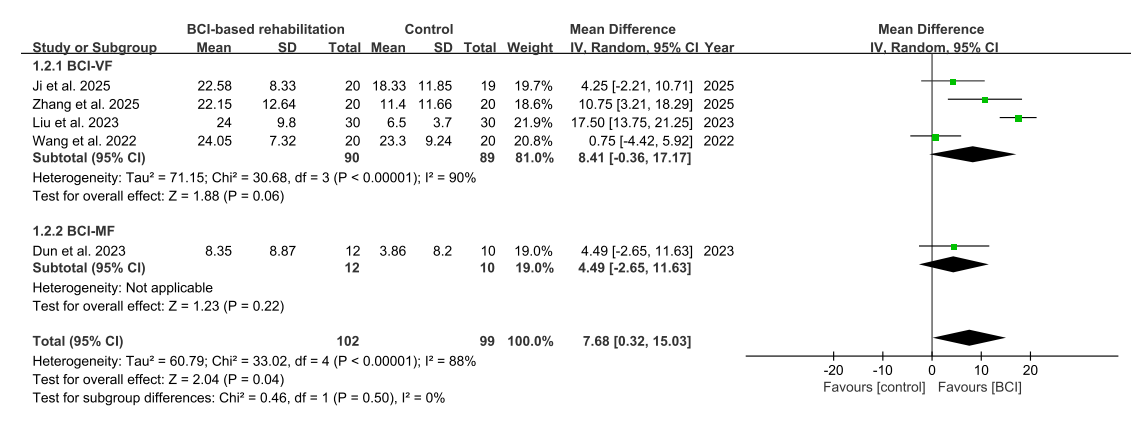


Figure S14 Forest plot for the pooled ES of BCI-based rehabilitation on activities of daily living in feedback modalities subgroup analysis


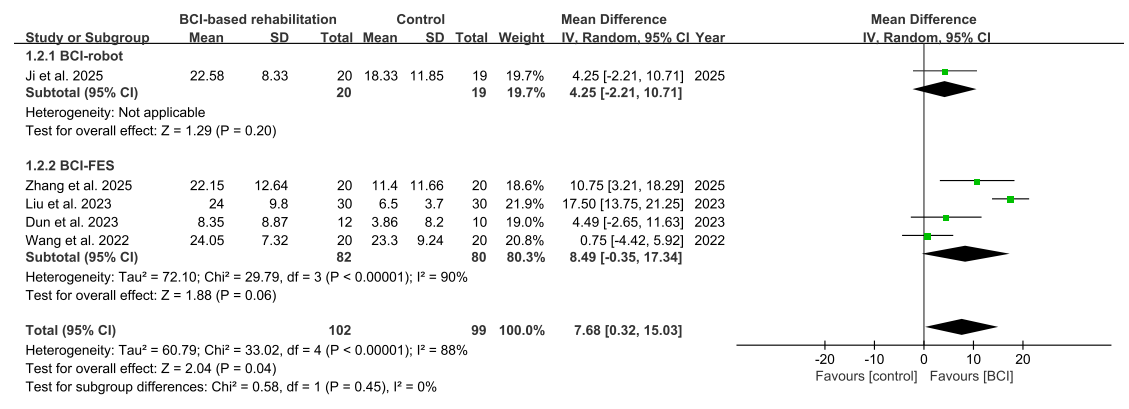


Figure S15 Forest plot for the pooled ES of BCI-based rehabilitation on activities of daily living in assistive technologies subgroup analysis


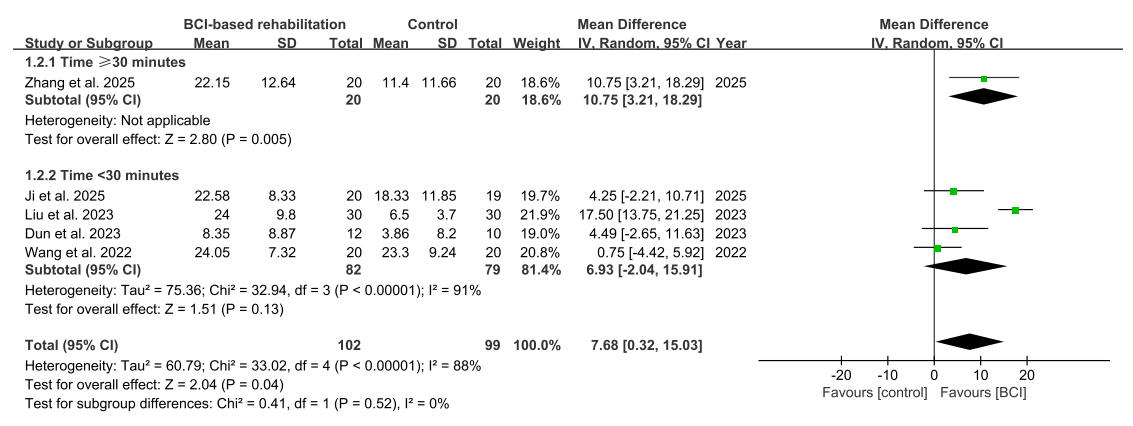


Figure S16 Forest plot for the pooled ES of BCI-based rehabilitation on activities of daily living in time subgroup analysis


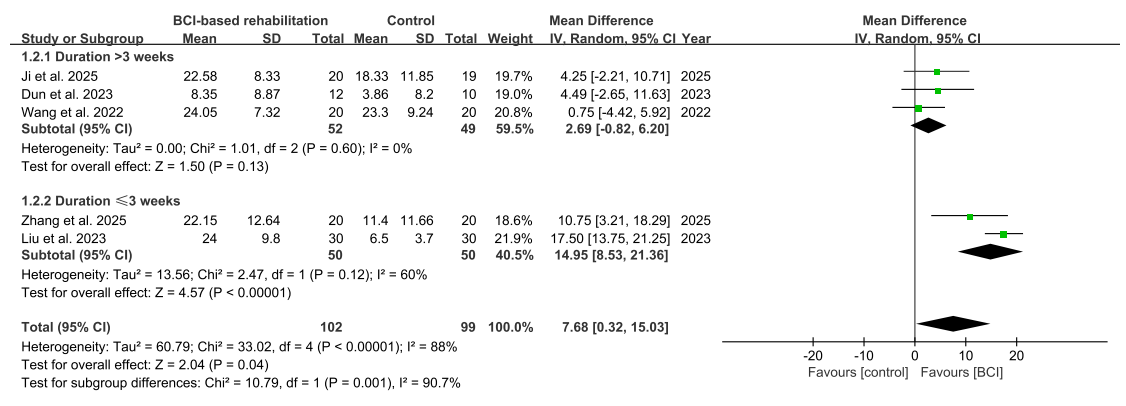


Figure S17 Forest plot for the pooled ES of BCI-based rehabilitation on activities of daily living in duration subgroup analysis

**Database search**

| **Embase** | |
| --- | --- |
| **Search** | **Query** |
| #1 | 'cerebrovascular accident'/exp |
| #2 | 'acute stroke':ti,ab,kw OR 'sub-acute stroke':ti,ab,kw OR 'early stroke':ti,ab,kw OR 'cerebrovascular accident':ti,ab,kw OR stroke:ti,ab,kw OR 'brain vascular accident':ti,ab,kw OR 'brain attack':ti,ab,kw OR 'cerebral stroke':ti,ab,kw OR 'apoplexy':ti,ab,kw OR 'hemiplegia':ti,ab,kw OR 'cva':ti,ab,kw |
| #3 | #1 OR #2 |
| #4 | 'brain-computer interface'/exp |
| #5 | 'brain-computer interface':ti,ab,kw OR 'bci':ti,ab,kw OR 'brain-machine interface':ti,ab,kw OR 'bmi':ti,ab,kw OR 'neural interface':ti,ab,kw OR 'neurotechnology':ti,ab,kw |
| #6 | #4 OR #5 |
| #7 | 'upper limb function':ti,ab,kw OR 'arm function':ti,ab,kw OR 'upper extremity function':ti,ab,kw OR 'upper limb mobility':ti,ab,kw OR 'Fugl-Meyer Assessment':ti,ab,kw OR 'fma':ti,ab,kw OR 'activities of daily living':ti,ab,kw OR 'adl':ti,ab,kw OR 'Modified Barthel Index':ti,ab,kw OR 'mbi':ti,ab,kw OR 'Functional Independence Measure':ti,ab,kw OR 'fim':ti,ab,kw OR 'adverse events':ti,ab,kw OR 'adverse effects':ti,ab,kw OR 'side effects':ti,ab,kw OR 'untoward effects':ti,ab,kw OR 'harmful effects':ti,ab,kw OR 'complications':ti,ab,kw |
| #8 | #3 AND # 6 AND #7 |
| #9 | #8 AND 'randomized controlled trial'/de |

| **CINAHL** | |
| --- | --- |
| **Search** | **Search Terms** |
| S1 | MH acute stroke OR MH sub-acute stroke OR MH early stroke OR MH cerebrovascular accident OR MH stroke OR MH brain vascular accident OR MH brain attack OR MH cerebral stroke OR MH apoplexy OR MH hemiplegia OR MH CVA |
| S2 | MH brain-computer interface OR MH BCI OR MH brain-machine interface OR MH BMI OR MH neural interface OR MH neurotechnology |
| S3 | MH upper limb function OR MH arm function OR MH upper extremity function OR MH upper limb mobility OR MH Fugl-Meyer Assessment OR MH FMA OR MH activities of daily living OR MH ADL OR MH Modified Barthel Index OR MH MBI OR MH Functional Independence Measure OR MH FIM OR MH adverse events OR MH adverse effects OR MH side effects OR MH untoward effects OR MH harmful effects OR MH complications |
| S4 | S1 AND S2 AND S3 |
| S5 | S4 AND MH random* control* trials |

| **Cochrane Library** | |
| --- | --- |
| **Search** | **Search Terms** |
| #1 | MeSH descriptor: stroke |
| #2 | (acute stroke OR sub-acute stroke OR early stroke OR cerebrovascular accident OR stroke OR brain vascular accident OR brain attack OR cerebral stroke OR apoplexy OR hemiplegia OR CVA):ab,ti,kw |
| #3 | #1 OR #2 |
| #4 | MeSH descriptor: brain-computer interface |
| #5 | (brain-computer interface OR BCI OR brain-machine interface OR BMI OR neural interface OR neurotechnology):ab,ti,kw |
| #6 | #4 OR #5 |
| #7 | (upper limb function OR arm function OR upper extremity function OR upper limb mobility OR Fugl-Meyer Assessment OR FMA OR activities of daily living OR ADL OR Modified Barthel Index OR MBI OR Functional Independence Measure OR FIM OR adverse events OR adverse effects OR side effects OR untoward effects OR harmful effects OR complications):ab,ti,kw |
| #8 | #3 AND # 6 AND #7 |
| #9 | #8 AND (random* control* trials):ab,ti,kw |

| **CNKI** **(English version)** | |
| --- | --- |
| **Search** | **Search Terms** |
| S1 | (Keyword：acute stroke) OR (Keyword：sub-acute stroke) OR (Keyword：early stroke) OR (Keyword：cerebrovascular accident) OR (Keyword：stroke) OR (Keyword：brain vascular accident) OR (Keyword：brain attack) OR (Keyword：cerebral stroke) OR (Keyword：apoplexy) OR (Keyword：hemiplegia) OR (Keyword：CVA) |
| S2 | (Keyword：brain-computer interface) OR (Keyword：BCI) OR (Keyword：brain-machine interface) OR (Keyword：BMI) OR (Keyword：neural interface) OR (Keyword：neurotechnology) |
| S3 | (Keyword：upper limb function) OR (Keyword：arm function) OR (Keyword：upper extremity function) OR (Keyword：upper limb mobility) OR (Keyword：Fugl-Meyer Assessment) OR (Keyword：FMA) OR (Keyword：activities of daily living) OR (Keyword：ADL) OR (Keyword：Modified Barthel Index) OR (Keyword：MBI) OR (Keyword：Functional Independence Measure) OR (Keyword：FIM) OR (Keyword：adverse events) OR (Keyword：adverse effects) OR (Keyword：side effects) OR (Keyword：untoward effects) OR (Keyword：harmful effects) OR (Keyword：complications) |
| S4 | S1 AND S 2 AND S3 |
| S5 | S4 AND (Keyword: randomized controlled trial) |

| **CNKI (Chinese version)** | |
| --- | --- |
| **检索** | **检索术语** |
| #1 | （关键词：急性卒中）或（关键词：亚急性脑卒中）或（关键词：早期脑卒中）或（关键词：脑血管意外）或（关键词：卒中）或（关键词：脑血管事故）或（关键词：脑发作）或（关键词：脑卒中）或（关键词：中风）或（关键词：偏瘫）或（关键词：CVA） |
| #2 | (关键词：脑机接口) 或 (关键词：BCI) 或 (关键词：脑机械接口) 或 (关键词：BMI) 或 (关键词：神经接口) 或 (关键词：神经技术) |
| #3 | (关键词：上肢功能) 或 (关键词：手臂功能) 或 (关键词：上肢运动功能) 或 (关键词：上肢活动能力) 或 (关键词：Fugl-Meyer 评估) 或 (关键词：FMA) 或 (关键词：日常生活活动能力) 或 (关键词：ADL) 或 (关键词：改良 Barthel 指数) 或 (关键词：MBI) 或 (关键词：功能独立性测量) 或 (关键词：FIM) 或 (关键词：不良事件) 或 (关键词：不良反应) 或 (关键词：副作用) 或 (关键词：不良影响) 或 (关键词：有害反应) 或 (关键词：并发症) |
| #4 | #1和 # 2和 #3 |
| #5 | #4 和（关键词：随机对照试验） |
